# Characterization and regulation of the Resistance-Nodulation-Cell Division-type multidrug efflux pumps MdtABC and MdtUVW from the fire blight pathogen *Erwinia amylovora*

**DOI:** 10.1186/1471-2180-14-185

**Published:** 2014-07-11

**Authors:** Daniel Pletzer, Helge Weingart

**Affiliations:** 1 School of Engineering and Science, Jacobs University Bremen, Campus Ring 1, 28759 Bremen, Germany

**Keywords:** Plant pathogen, Fire blight, Erwinia amylovora, Multidrug efflux, RND transporter, MdtABC, MdtUVW, BaeR, CpxR

## Abstract

**Background:**

The Gram-negative bacterium *Erwinia amylovora* is the causal agent of the devastating disease fire blight in rosaceous plants such as apple, pear, quince, raspberry, and cotoneaster. In order to survive and multiply in a host, microbes must be able to circumvent the toxic effects of antimicrobial plant compounds, such as flavonoids and tannins. *E. amylovora* uses multidrug efflux transporters that recognize and actively export toxic compounds out of the cells. Here, two heterotrimeric resistance-nodulation-cell division (RND)-type multidrug efflux pumps, MdtABC and MdtUVW, from *E. amylovora* were identified. These RND systems are unusual in that they contain two different RND proteins forming a functional pump.

**Results:**

To find the substrate specificities of the two efflux systems, we overexpressed the transporters in a hypersensitive mutant lacking the major RND pump AcrB. Both transporters mediated resistance to several flavonoids, fusidic acid and novobiocin. Additionally, MdtABC mediated resistance towards josamycin, bile salts and silver nitrate, and MdtUVW towards clotrimazole. The ability of the *mdtABC*- and *mdtUVW*-deficient mutants to multiply in apple rootstock was reduced. Quantitative RT-PCR analyses revealed that the expression of the transporter genes was induced during infection of apple rootstock. The polyphenolic plant compound tannin, as well as the heavy metal salt tungstate was found to induce the expression of *mdtABC*. Finally, the expression of the *mdtABC* genes was shown to be regulated by BaeR, the response regulator of the two-component system BaeSR, a cell envelope stress response system that controls the adaptive responses to changes in the environment.

**Conclusions:**

The expression of MdtABC and MdtUVW is induced during growth of *E. amylovora in planta*. We identified the plant polyphenol tannin as inducer of *mdtABC* expression. The reduced ability of the *mdtABC*- and *mdtUVW*-deficient mutants to multiply in apple rootstock suggests that the efflux pumps are involved in resistance to plant antimicrobials, maybe including flavonoids, which were identified as substrates of both pumps. Furthermore, we found that the *mdtABC* operon belongs to the regulon of the two-component regulator BaeR suggesting a role of this RND transporter in the cell envelope stress response of *E. amylovora*.

## Background

Fire blight, caused by the Gram-negative enterobacterium *Erwinia amylovora*, is a devastating disease of rosaceous plants in the subfamily Maloideae that has global economic importance for apple and pear production [1]. Typical symptoms include flower necrosis, immature fruit rot, shoot curvature (shepherd’s crook), blackened leaves (these generally remain attached to the plant), bacterial ooze secretion, and cankers on woody tissues.

Production of phenolic compounds with antimicrobial activity (e.g. flavonoids, tannins) is an important part of the plant defense repertoire to limit the spread of pathogens [2]. Successful phytopathogens must be able to circumvent the toxic effects of antimicrobial plant compounds. An important resistance mechanism of bacteria against plant-borne antimicrobials involves efflux of these compounds by so-called multidrug transporters. Among the transporter families containing multidrug efflux transporters, the resistance-nodulation-cell division (RND) family has been identified as the most relevant in terms of resistance to clinically important agents in Gram-negative bacteria [3,4]. Members of the RND family are able to recognize and expel a broad range of antimicrobials from the cell [5,6]. The RND-type efflux pump AcrAB has been shown to be involved in virulence of *E. amylovora* conferring resistance to plant-borne antimicrobial compounds like apple phytoalexins [7].

The functional RND-type efflux pump is a tripartite complex, consisting of the RND-type transporter protein located in the inner membrane, a periplasmic membrane protein, and an outer membrane channel [5]. The inner membrane RND transporter is a homotrimer that uses the proton gradient as an energy source [8,9]. Associated with this homotrimeric structure is the so-called ‘rotating mechanism’, a conformational change in the periplasmic core domain to export drug molecules [10].

However, not all members of the RND family follow a homotrimeric organization. For example, the RND-family efflux system MdtABC from *E. coli* possesses two different RND transporters, MdtB and MdtC, which are co-transcribed in an operon with the membrane fusion protein MdtA. It has previously been shown that the functional pump consists of a heteromultimeric unit formed by two subunits of MdtB and one subunit of MdtC [11]. Furthermore, it has been suggested that MdtC is involved in binding and transport of drugs and that MdtB presumably induces the conformational change needed for transport using the proton translocation as an energy source [12].

Previous genetic studies have demonstrated that the deletion of the heteromultimeric RND pump MdtABC abolishes the resistance of *E. coli* and *Salmonella enterica* to β-lactams, novobiocin, SDS, and bile salts [13-15]. Moreover, MdtABC has been implicated in detoxification of heavy metals, in particular, in resistance to zinc, copper and tungstate [15-17].

In *E. coli*, the expression of the multidrug efflux pump MdtABC is regulated by two stress response systems, Bae and Cpx. The BaeSR and CpxARP two-component signal transduction systems respond to damage of the cell envelope; however, they differ with regard to specific inducers. The BaeSR regulon responds to a wide range of environmental stresses, including spheroplast formation, overexpression of the PapG pilin under conditions leading to misfolding, and exposure to indole, tannins, flavonoids, tungstate, and zinc [16,18-20]. The small core regulon of BaeSR includes the BaeSR two-component system itself, the RND-type transporters AcrD and MdtABC, and the periplasmic chaperone Spy [19]. The CpxRA regulon of *E. coli* contains hundreds of genes, including periplasmic protein folding and degrading factors, peptidoglycan metabolic enzymes, inner membrane proteins and regulators, and envelope-localized protein complexes, such as pili and flagella [21-23]. The Cpx system comprises the sensor histidine kinase CpxA, the cytoplasmic response regulator CpxR, and CpxP, a periplasmic inhibitor of CpxA [24]. The Cpx pathway is activated by a variety of stresses within the bacterial cell envelope, e.g., alkaline pH, alterations to the composition of the inner membrane, overexpression of misfolded envelope proteins, and adhesion to hydrophobic surfaces sensed by the lipoprotein NlpE [23,25]. Hirakawa et al. [26] reported that the overexpression of BaeR and CpxR causes up-regulation of RND pumps AcrD and MdtABC in *E. coli*. Analysis of the induction of multidrug transporter genes by indole revealed that the CpxR-mediated induction of *acrD* and *mdtABC* genes depends on the BaeSR two-component system [27]. These results indicate that BaeR is a primary regulator, and CpxR enhances the effect of BaeR.

The aim of this study was to characterize the heteromultimeric RND-type multidrug efflux pumps MdtABC and MdtUVW from *E. amylovora* Ea1189 and to determine the role of the two-component system regulators BaeR and CpxR in regulation of these efflux systems.

## Results

### Computational analysis of RND-type transporters from *E. amylovora* Ea1189

**Figure 1:**
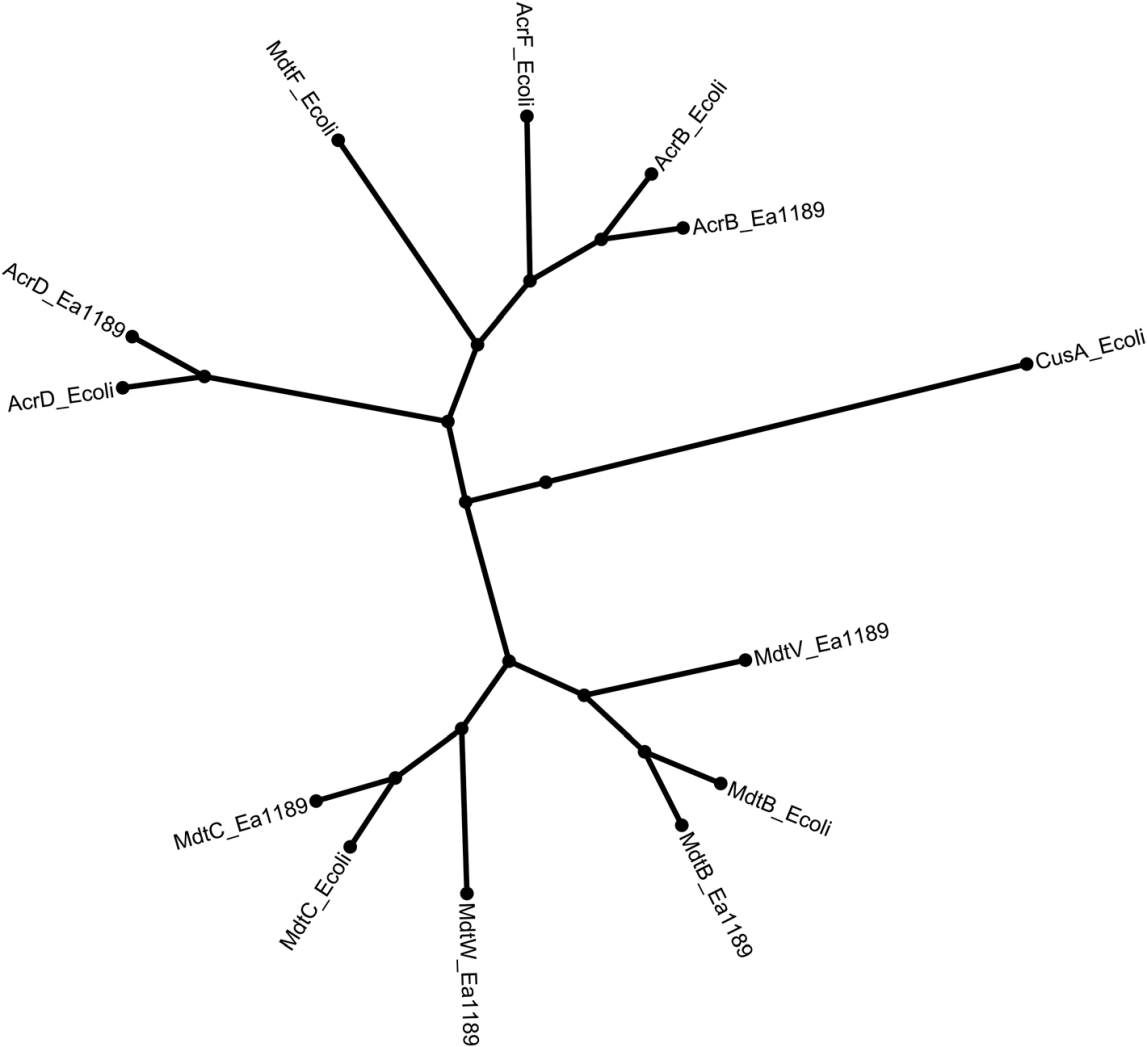
**Phylogenetic tree of the RND transporters AcrB, AcrF, AcrD, CusA, MdtF, MdtB, and MdtC from *E. coli *MG1655 and AcrB, AcrD, MdtB, MdtC, MdtV and MdtW from *E. amylovora *Ea1189.** Multiple sequence alignment was performed using Clustal Omega [28] and the result visualized by FigTree [29].

Analysis of the genome sequence of *E. amylovora* revealed the presence of two, hitherto not characterized, operons encoding heterotrimer-type RND-type efflux pumps. Both show homology to the *mdtABC* operon of *E. coli*. The *mdtABC* genes of *E. coli* encode an RND system that is unusual in that it contains two different RND pump genes, *mdtB* and *mdtC*, in addition to the membrane fusion protein gene, *mdtA*[11]. One of the heterotrimer-type RND efflux systems of *E. amylovora* shows a high degree of homology to the *E. coli* MdtABC transporter as depicted in the phylogenetic tree in Figure 1. We accordingly propose to name the operon encoding this transporter as *mdtABC*. The second, more distantly related operon was named *mdtUVW*.

A sequence alignment showed that the RND pump MdtB from *E. amylovora* Ea1189 shares 59% identity with MdtV and 81% identity with MdtB from *E. coli* (Additional file 1). MdtV shares only 61% identity with MdtB from *E. coli*. The RND pump MdtC from *E. amylovora* shares 54% identity with MdtW and 74% identity with MdtC from *E. coli*. MdtW shares 56% identity with MdtC from *E. coli*. Furthermore, *E. coli* possesses an additional gene, *iceT* (formerly known as *mdtD*), encoding an iron citrate efflux transporter of the major facilitator superfamily, within the *mdtABC* operon [30]. No homologue of the *iceT* is present in the genome of *E. amylovora*.

Analysis of the up- and downstream regions flanking the *mdtABC* operons from *E. amylovora* and *E. coli* revealed the presence of the *baeSR* genes located downstream of the *mdtABC* operons in both organisms (Additional file 2). The two-component system BaeSR is involved in a unique envelope stress response in *E. coli*[18].

Topological analysis of the RND proteins MdtB, MdtC, MdtV, and MdtW from *E. amylovora* using the TOPCONS software [31] predicted 12 transmembrane-spanning domains (TMDs) and two large periplasmic loops between TMD 1 and 2 and TMD 7 and 8 for all four transporters (Additional file 3). This transmembrane organization is typical for members of the RND family [32,33].

### Phenotypic characterization of the *mdtABC* and *mdtUVW* mutants

In order to investigate the role of the RND-type multidrug efflux pumps MdtABC and MdtUVW in antibiotic resistance and to identify pump-specific substrates, antimicrobial susceptibility tests of the wild type and the corresponding mutants were performed. However, deletion of the *mdtABC* and *mdtUVW* operon, respectively, resulted in no changes in sensitivity to all tested antimicrobial agents including plant-derived antimicrobials, antibiotics, dyes, and heavy metals (Table 1).

**Table 1:** Antimicrobial susceptibility profiles

**Drug**	**MIC (μg/ml)**^**a**^					
	**Ea1189**	**Ea1189.ΔmdtABC**	**Ea1189.ΔmdtUVW**	**Ea1189-3**	**Ea1189-3 pBlueSK.mdtABC**	**Ea1189-3 pBlueKS.mdtUVW**
**Phytochemicals**						
Apple extract	> 100000	> 100000	> 100000	25000	50000	25000
Tannin	625	625	625	1250	**5000**	1250
Flavones						
Apigenin	> 2500	> 2500	> 2500	31.2	**125**	**> 1000**
Daidzein	1000	1000	1000	156	**1000**	**1000**
Genistein	> 1000	> 1000	> 1000	62.5	125	**> 1000**
Kaempferol	> 5000	> 5000	> 5000	250	**1000**	**1000**
Luteolin	> 1000	> 1000	> 1000	12.5	25	**> 100**
Myricetin	2500	2500	2500	1250	1250	1250
Naringenin	1000	1000	1000	250	250	**1000**
Quercitin	5000	5000	5000	2500	2500	5000
Orobol	1000	1000	1000	31.2	31.2	**1000**
**β-Lactams**						
Ampicillin	312	312	312	25	50	50
Carbenicillin	312	312	312	50	50	50
Cefepime	12.5	12.5	12.5	0.16	0.31	0.16
Cefoxitin	62.5	62.5	62.5	25	25	25
Cefsulodin	156	156	156	50	50	50
Ceftazidime	1000	1000	1000	6.2	6.2	6.2
Cloxacillin	2500	2500	2500	25	25	25
Oxacillin	1250	1250	1250	12.5	25	25
Ticarcillin	250	250	250	50	50	50
**Aminoglycosides**						
Amikacin	3.1	3.1	3.1	5	5	5
Gentamicin	2.5	2.5	2.5	2.5	2.5	2.5
Kanamycin	6.2	6.2	6.2	> 100	> 100	> 100
Neomycin	3.1	3.1	3.1	> 100	> 100	> 100
Streptomycin	3.1	3.1	3.1	5	5	5
Tobramycin	1.2	1.2	1.2	5	5	5
**Antibiotics**						
Chloramphenicol	3.1	3.1	3.1	1.5	1.5	1.5
Erythromycin	1.2	1.2	1.2	0.3	0.3	0.6
Fosfomycin	1250	1250	1250	156	312	156
Fusidic acid	250	250	250	6.2	**62.5**	**100**
Josamycin	250	250	250	6.2	**50**	12.5
Lincomycin	1250	1250	1250	78	78	78
Norfloxacin	0.13	0.13	0.13	0.03	0.03	0.06
Novobiocin	125	125	125	6.2	**100**	**100**
Rifampicin	50	50	50	31.2	31.2	50
Tetracycline	2.5	2.5	2.5	0.63	1.25	1.25
Trimethoprim	625	625	625	625	625	625
**Dyes**						
Acriflavine	31.2	31.2	31.2	25	25	25
Ethidium bromide	500	500	500	6.2	6.2	6.2
**Antimicrobials**						
Bile	5000	5000	5000	625	**2500**	1250
Clotrimazole	> 1000	> 1000	> 1000	6.2	6.2	**> 100**
Indole	625	625	625	625	625	625
SDS	625	625	625	125	125	250
**Heavy metals**						
Cadmium acetate	25	25	25	25	25	25
Copper sulfate	1250	1250	1250	1250	1250	1250
Silver nitrate	6.2	6.2	6.2	6.2	**25**	6.25
Sodium tungstate	125000	125000	125000	62500	62500	62500
Zinc sulfate	312	312	312	312	625	312

^a^MIC values were determined by the 2-fold dilution assay in three or more independent experiments with similar results. Boldface numbers indicate a more than 2-fold higher MIC. ND, not determined.

MIC values were determined from an *E. amylovora* wild-type strain, *mdtABC* mutant*,* and *mdtUVW* mutant, as well as from an *acrB* mutant Ea1189-3 complemented with MdtABC-overexpression plasmid pBlueSK.mdtABC or MdtUVW-overexpression plasmid pBlueKS.mdtUVW-ext.

### Effect of *mdtABC* and *mdtUVW* overexpression on multidrug resistance in an *acrB*-deficient mutant of *E. amylovora*

Since disruption of the *mdtABC* and *mdtUVW* operons did not cause hypersusceptibility to any of the tested compounds, possibly due to their low expression during cellular growth or due to the activity of the highly expressed AcrAB efflux pump, overexpression of the *mdtABC* and *mdtUVW* operons from high-copy plasmids in an *acrB*-deficient mutant was achieved. Different plasmids expressing the RND operons under control of three different promoters were generated: *lac* promoter (P_lac_), native promoter (P_mdtABC_ or P_mdtUVW_), and dual promoter (*lac* promoter and native promoter). To investigate the role of the RND pumps in β-lactam resistance, the *bla* gene of the high-copy plasmid pBlueScript II KS(+) and pBlueScript II SK(+) used for overexpression of the *mdtABC* and *mdtUVW* operons was replaced by a Sm/Sp resistance cassette (Additional file 4). All constructs were mobilized into the *acrB*-deficient mutant Ea1189-3 and the sensitivity of the transformants to various substrates was determined (Table 1).

Our initial data indicated that expression of the RND operons from different promoters had a strong influence on the MIC values. Therefore, we utilized qRT-PCR to analyze the mRNA expression from the different constructs and found that the expression of the *mdtABC* operon driven by P_lac_ (40-fold) was 10 times higher than the expression driven by the dual promoter (4-fold). Interestingly, there was almost no increased expression of the *mdtABC* operon from its native promoter (2-fold). Although we detected minor MIC changes after expression of the *mdtABC* operon from its native promoter and dual promoter, respectively, expression driven by P_lac_ resulted in the highest changes in MIC values. In contrast, the expression levels of the *mdtUVW* operon from P_lac_ or from its native promoter were similar low (2- to 4-fold). The expression of the *mdtUVW* operon from the dual promoter was much higher (18-fold), however, did not result in MIC changes. Interestingly, only with the expression from the native promoter, we were able to detect minor changes in MIC values.

The expression of *mdtABC* from a high-copy vector under the control of the *lac* promoter, in an *acrB*-deficient mutant, resulted in increased resistance to tannin (4-fold), apigenin (4-fold), daidzein (8-fold), kaempferol (4-fold), fusidic acid (8-fold), josamycin (8-fold), novobiocin (16-fold), bile salts (4-fold) and silver nitrate (4-fold) (Table 1). The expression of *mdtUVW* from a high-copy vector under the control of its native promoter increased the MICs of several flavonoids including apigenin (>32-fold), daidzein (8-fold), genistein (>16-fold), kaempferol (4-fold), luteolin (>8-fold), naringenin (4-fold), and orobol (32-fold), and of the antibiotics fusidic acid (16-fold), novobiocin (16-fold) and clotrimazole (>16-fold) (Table 1).

### RND-type efflux pump expression during cellular growth

The relative mRNA transcript abundance of *mdtA* and *mdtU* from *E. amylovora* Ea1189 during cellular growth was determined by quantitative RT-PCR. Therefore, total RNA was isolated at distinct optical densities (OD_600_ of 0.5, 1.0, 1.5 and 2.0) and the expression normalized to the highest expression of each transcript. The mRNA transcript levels of *mdtA* and *mdtU* were constant but low during growth in LB medium as determined by C_t_ values (Additional file 5). Analysis of transcriptional fusions between the promoter regions of the efflux pumps and the reporter gene *egfp* supported these results. Fluorescence measurements showed that the activity of the *mdtABC* and *mdtUVW* promoters were 3- to 5-fold lower than the activity of the *acrA* promoter throughout growth in LB broth.

### Effect of substrate exposure on *mdtABC* and *mdtUVW* expression

To investigate whether antimicrobials effect the expression of *mdtABC* or *mdtUVW* in *E. amylovora*, transcriptional fusions between the *mdtABC* upstream region and *mdtUVW* upstream region, respectively, to the *egfp* gene were constructed, yielding plasmids pBBR.mdtABC-Pro.egfp and pBBR.mdtUVW-Pro.egfp.

Antimicrobial compounds were added to the plasmid-harboring cells by the 2-fold dilution method in 96-well plates and EGFP fluorescence was determined after 24 hours. Only fluorescence values from substrate concentrations that did not inhibit bacterial growth were considered. Potential inducers of gene expression (49 compounds have been tested), showing higher fluorescence than the remaining dataset, were identified as fusaric acid for the *mdtABC* promoter and copper sulphate for the *mdtUVW* promoter (data not shown).

In addition, we analyzed the expression of *mdtA* and *mdtU* by qRT-PCR after 2 hours induction with several antimicrobials. Tested compounds were phloretin, naringenin, mycricetin, methanolic and acetonic apple extract, indole, paraquat, phenolic acids, gallic acid, tannin, indole-3-acetic acid, and the metals iron, copper, zinc, and tungstate. Our data showed an induction of *mdtA* by tannin (4.4-fold) and tungstate (2.4-fold), while expression of *mdtU* was not induced by the tested compounds (Table 2). Furthermore, we observed that the regulatory gene *baeS* was 2.8-fold induced by tannin (data not shown).

**Table 2:** **Relative fold-changes of *mdtA *** **and *mdtU *** **mRNA transcripts in *E. amylovora *** **Ea1189 after 2 h of incubation with transporter substrates as determined by qRT-PCR**^***a***^

	***mdtA***^***b***^	***mdtU***
Methanolic apple extract (1 μl/ml)	1.2	1.2
Acetonic apple extract (10 μl/ml)	1.2	1.5
Tannin (0.5 mg/ml)	**4.4**	1.7
Phloretin (4 μg/ml)	0.8	0.9
Naringenin (8 μg/ml)	1.1	1.1
Myricetin (10 μg/ml)	0.8	0.7
Indole (2 mM)	1.0	0.7
Paraquat (0.2 mM)	0.9	1.3
Phenolic acids^*c*^ (0.078 mM)	0.9	0.7
Gallic acid (1 mg/ml)	1.4	1.5
Indole-3-acetic acid (2 mM)	0.8	0.7
Iron sulphate (1 mM)	0.9	0.8
Copper sulphate (1 mM)	1.1	1.4
Zinc sulphate (1 mM)	1.1	1.4
Sodium Tungstate (20 mM)	**2.4**	1.2

^*a*^Total RNA was isolated from bacterial cells incubated for 2 hours with transporter substrates in LB broth. Transcript abundance was determined by quantitative RT-PCR and compared to RT-PCR signal from cells grown in LB broth containing only the solvent of the respective substrate.

^*b*^Boldface values indicate an increase of at least 2-fold. Represented data values are the means of at least three replicates.

^*c*^A mixture of 0.078 mM salicylic acid and 0.078 mM benzoic acid was used.

### Regulation of the RND-type pumps MdtABC and MdtUVW in *E. amylovora*

Several studies suggest a connection between cell envelope stress responses and expression of multidrug efflux systems. In *E. coli*, the two-component signal transduction systems BaeSR and CpxARP respond to damage of the cell envelope and have been shown to regulate the expression of drug exporter genes including the RND efflux pump MdtABC [13,14,27]. Since a BLAST search revealed the presence of homologous systems in the genome sequence of *E. amylovora* (Additional file 6), it prompted us to investigate whether the response regulators BaeR and CpxR control expression of the MdtABC and MdtUVW transporters in *E. amylovora* Ea1189.

**Figure 2:**
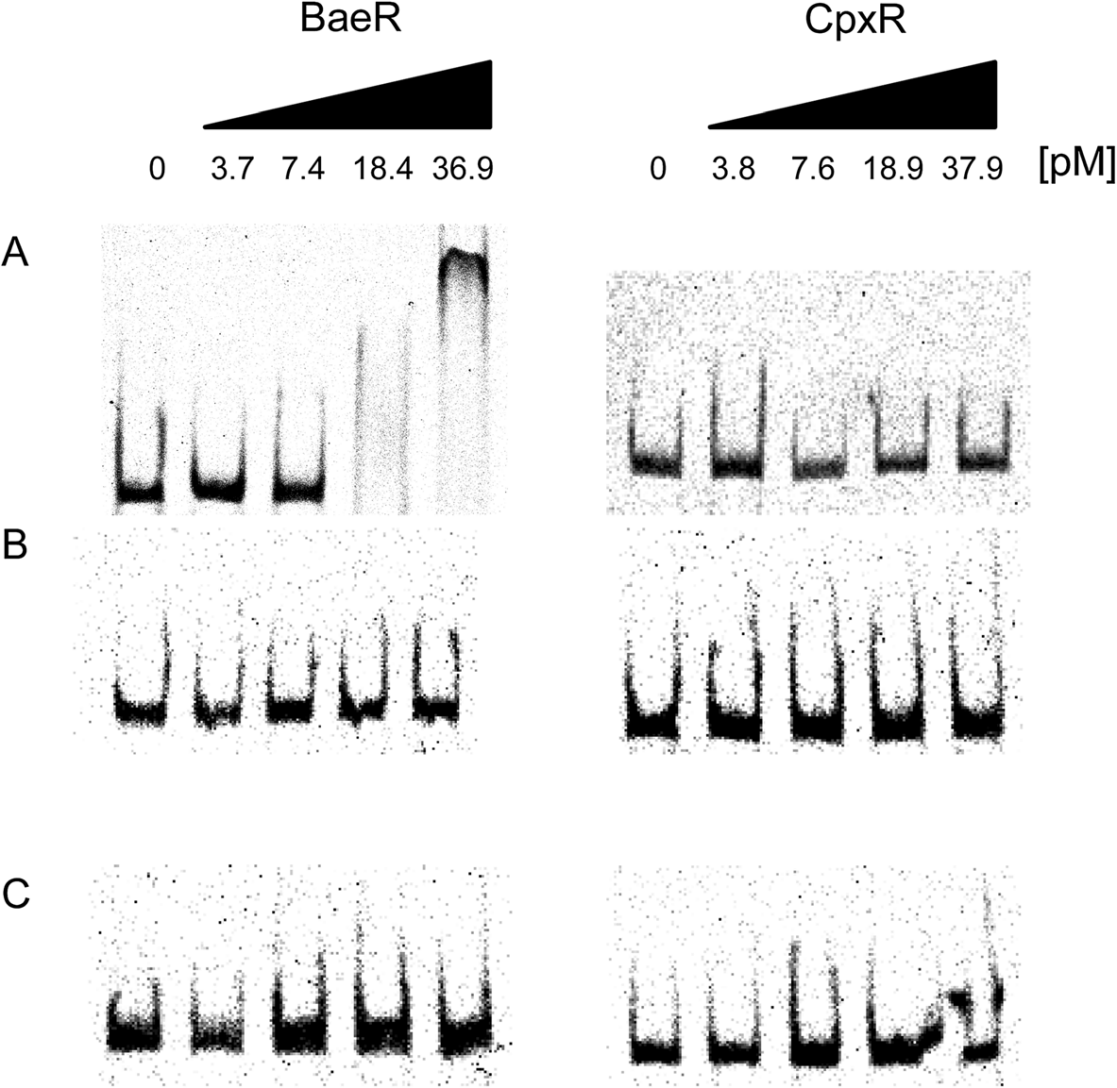
**Electrophoretic mobility shift analysis of BaeR and CpxR interaction with Cy5-labeled DNA fragments.** DNA fragments contain the promoter regions of **(A)***mdtABC* (290 bp), **(B)***mdtUVW* (276 bp) and **(C)** a fragment from within the *tolC* gene (248 bp) as control. Approximately 0.3 pmol of the DNA fragments were incubated with increasing amounts of His-tag purified BaeR and CpxR, respectively (indicated at the top of the panel). The DNA-protein complexes were separated on 4% non-denaturing polyacrylamide gels.

To test whether BaeR or CpxR bind to the promoter regions of *mdtABC* or *mdtUVW in vitro*, an electrophoretic mobility shift assay (EMSA) was performed. DNA fragments used in the EMSA were the Cy5-labeled upstream regions of *mdtABC* (290 bp), *mdtUVW* (276 bp) and as control, a fragment located within the *tolC* gene (248 bp). The DNA fragments were incubated with increasing amounts of purified BaeR or CpxR protein in the presence of nonspecific competitor DNA (Salmon sperm) (Figure 2). The purified BaeR protein showed binding to the upstream region of *mdtABC* with increasing concentrations (Figure 2A). However, no interaction between BaeR and the *mdtUVW* promoter region was observed. Furthermore, no interaction between CpxR and the *mdtABC* or *mdtUVW* promoter region was detected. *In vitro* phosphorylation of the purified protein using acetyl phosphate did not lead to a binding of CpxR to one of the DNA fragments. Phosphorylation of BaeR enhanced the binding to the *mdtABC* promoter region about 2-fold.

### Induction of *mdtABC and mdtUVW* expression by the response regulators BaeR and CpxR

Owing to the interaction between the *mdtABC* promoter region and BaeR in the EMSA, we investigated whether overexpression of BaeR and CpxR may induce the expression of efflux pump genes *in vivo*. Therefore, *baeR* and *cpxR* were cloned under control of an arabinose-inducible promoter into plasmid pBAD24 and the constructs were mobilized into Ea1189 wild-type cells. Analysis of the expression levels using qRT-PCR identified a 13-fold induction of *baeR* and a 49-fold induction of *cpxR* in Ea1189 cells harboring the respective plasmids (Table 3). We have previously reported that overexpression of BaeR induces the expression of the RND pump gene *acrD* about 4-fold, while the expression of *acrA* and *tolC* was not affected [34]. In this study we found that overexpression of BaeR increased the expression of *mdtABC* more than 20-fold (Table 3). However, no induction of the MdtUVW pump was observed. The qRT-PCR results correlate well with the observed interaction of BaeR in the EMSA, indicating a specific binding of BaeR to the promoter region of *mdtABC*.

**Table 3:** **Relative fold-changes of mRNA transcripts of RND-type efflux pumps and the outer membrane protein TolC in *E. amylovora *** **Ea1189 harboring plasmids pBAD.baeR and pBAD.cpxR, respectively**^***a***^

	***acrA***	***acrD***^***b***^	***mdtA***	***mdtU***	***tolC***	***baeR***	***cpxR***
pBAD.baeR	0.8	**3.8**	**20.5**	0.8	0.7	**13.3**	0.9
pBAD.cpxR	1.2	1.5	0.9	0.9	1.2	0.9	**49.0**

^*a*^Total RNA was isolated from bacterial cells grown in LB broth at 28°C and induced with 1% L-arabinose for 1 hour. Transcript abundance was determined by quantitative RT-PCR and compared to RT-PCR signal of uninduced cultures.

^*b*^Boldface values indicate an increase of at least 2-fold. Represented data values are the means of at least three replicates.

In contrast, overexpression of the CpxR response regulator did not alter the expression of any multidrug efflux pump gene in *E. amylovora* (Table 3).

### Transcriptional analysis of *mdtA* and *mdtU in planta*

In order to analyze the expression of *mdtA* and *mdtU in planta*, Ea1189 was inoculated into shoot tips of apple rootstocks MM106 as well as onto immature pear fruit slices. Bacteria were re-isolated from immature pear fruit slices 12 hours after inoculation and 1, 3 and 7 days, respectively, after inoculation from apple shoot tips. Infected plant tissue was macerated and total RNA isolated from recovered cells to determine the transcript abundances of *mdtA* and *mdtU* by quantitative RT-PCR. RT-PCR signals of recovered bacteria were compared with RT-PCR signals of Ea1189 cells grown in LB broth to an OD_600_ of 0.5 (Table 4). Analysis of relative fold changes in mRNA transcripts showed that the expression of *mdtA* and *mdtU* increased 1.8- and 1.7-fold, respectively, on immature pear fruits. Our results further demonstrate that expression of *mdtA* was high throughout the seven monitored days (37- to 59-fold increased when compared to data obtained from growth in LB medium), which might also be due to the very low expression in LB broth during the cell cycle. In connection with the *mdtA* expression, the *mdtU* expression was about 3-fold induced within the first three days after infection of apple rootstock and returned to the base level after seven days.

**Table 4:** **Relative fold-changes of *mdtA*, *mdtU*, *baeS *** **and *cpxR *** **mRNA transcripts after inoculation of *E. amylovora *** **Ea1189 on apple rootstocks MM106 and immature pear fruit slices, respectively**^***a***^

**Gene**	**Apple rootstock**			**Immature pear**
	1 dpi^*b*^	4 dpi	7 dpi	12 hpi^*c*^
*mdtA*	**58.7**^*d*^	**36.7**	**54.2**	1.7
*mdtU*	**2.6**	**3.4**	1.4	1.8

^*a*^Total RNA was isolated from bacterial cells recovered from infected plant tissues. Transcript abundance was determined by quantitative RT-PCR and compared to RT-PCR signals from cells grown in LB broth to an OD_600_ of 0.5.

^*b*^Bacteria were re-isolated from infected shoots of apple rootstock 1, 4 and 7 days post inoculation (dpi).

^*c*^Bacteria were re-isolated from infected immature pears 12 hours post inoculation (hpi).

^*d*^Boldface values indicate an increase of at least 2-fold.

### Virulence of *E. amylovora* efflux pump mutants on apple rootstocks and immature pear fruits

**Figure 3:**
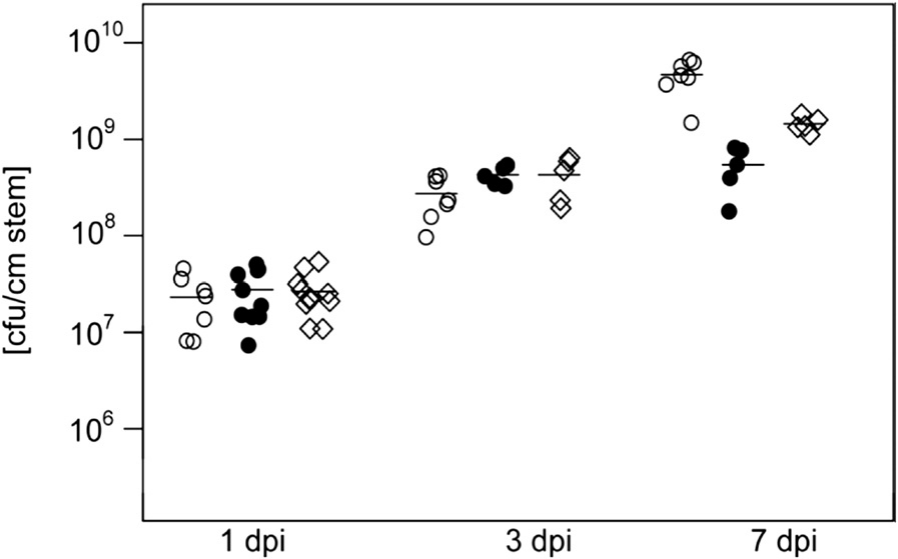
**Virulence assay on apple rootstock MM106.** Bacteria were inoculated by prick technique in the shoot tips with an inoculum of 5 × 10^6^ CFU/shoot. Establishment of a population of *E. amylovora* Ea1189 wild type (○), *mdtUVW*-deficient mutant (●) and *mdtABC*-deficient mutant (◊) was determined 1, 3 and 7 days post inoculation (dpi), respectively. A minimum of 5 plant shoot tips were inoculated. Data points represent the CFU/cm stem per shoot tip. The horizontal line through the points of a dataset indicates the mean value of the replicates. Average values and standard deviations are listed in Additional file 7.

Apple rootstocks MM106 were used to monitor the development of disease symptoms after infection with *E. amylovora* Ea1189 and corresponding RND-type efflux pump mutants Ea1189.ΔmdtABC and Ea1189.ΔmdtUVW. Seven days after infection all injected shoots showed typical disease symptoms including the shepherd’s crook-like bending, tissue necrosis and ooze formation. In order to study the establishment of bacterial populations within the tissue, samples were taken 1, 3 and 7 day(s) post inoculation and CFUs per stem determined. Three days after inoculation the wild type and the mutants showed similar growth. However, after seven days the population size of the *mdtABC* and *mdtUVW* mutant was 5- and 10-times lower, respectively, than the population size of the wild type. These results indicate that both RND-type pumps contribute to the ability of *E. amylovora* to survive and multiply in apple rootstock MM106 (Figure 3, Additional file 7).

Since *E. amylovora* is also able to infect pears, we used immature pear fruits to study the impact of the RND-pumps AcrB, MdtABC and MdtUVW on virulence. Fruits were infected with the wild type and the mutants and incubated at 28°C for 6 days and at 18°C for 14 days. However, except for the *acrB* mutant, no differences between the wild type and the mutants were observed (Additional file 8).

## Discussion

The highly virulent plant pathogen *E. amylovora* Ea1189 possesses four RND-type multidrug efflux transporters: AcrAB, AcrD, MdtABC, and MdtUVW. AcrAB has previously been shown to be involved in resistance against a broad range of structurally unrelated compounds including plant-borne antimicrobials [7]. AcrD has been characterized as efflux transporter conferring resistance to a limited number of amphiphilic compounds [34]. The RND transporter AcrB and AcrD exist as homotrimers in the inner membrane and conduct drug transport by the functionally rotating mechanism [35]. The genome sequence of *E. amylovora* contains two additional operons encoding RND-type efflux transporters, *mdtABC* and *mdtUVW*, with homology to the MdtABC transporter from *E. coli*. The heterotrimeric MdtB_2_C complex is the functional form of the *E. coli* transporter. MdtC is likely involved in substrate binding and transport, whereas the two copies of MdtB use proton translocation as energy source to induce the conformational change needed for drug transport [12]. This work aimed to characterize the heterotrimeric RND transporters MdtABC and MdtUVW from *E. amylovora* Ea1189, analyze the regulation of their expression, and identify their role in virulence.

Since deletion of the *mdtABC* or *mdtUVW* operon did not alter the cellular drug resistance profile of the respective *E. amylovora* mutants (Table 1), we overexpressed the efflux operons in the *E. amylovora* mutant Ea1189-3 defective in the major multidrug efflux pump AcrB. Overexpression of the MdtABC transporter in *E. amylovora* Ea1189-3 led to increased resistance towards three flavonoids (apigenin, daidzein, kaempferol), tannin, fusidic acid, josamycin, novobiocin, bile salts and silver nitrate. On the other hand, overexpression of the MdtUVW transporter led to increased resistance towards several flavonoids (apigenin, daidzein, genistein, kaempferol, luteolin, naringenin, orobol), fusidic acid, novobiocin and clotrimazole. Additionally, we observed a slight increase in resistance of the MdtABC-overexpressing strain towards acetonic apple leaf extracts (Table 1). The MdtABC and MdtUVW pumps from *E. amylovora* share overlapping substrate specificities with the MdtABC pumps from *E. coli* and *S. enterica*[13,19,36]. Shared substrates are novobiocin, bile salts and flavonoids suggesting that these compounds may resemble the natural substrates of the pumps.

The intrinsic resistance of *E. amylovora* towards flavonoids and tannin, mediated by the efflux pumps MdtABC and MdtUVW, is interesting since an increased production of polyphenols has been identified as defense mechanism of plants against fungal and bacterial infections [37,38]. Flavonoids occur widely in plants, are biologically important and are chemically diverse groups of secondary metabolites that possess a wide range of biological activities, including defense against pathogens [37]. Inducible biosynthesis of 3-deoxyflavonoid has been demonstrated to be accompanied by increased resistance of apple and pear leaves to fire blight infections [39]. To survive and multiply in its host plants, *E. amylovora* must be able to circumvent the toxic effect of these antimicrobial phytoalexins. We have previously demonstrated that the RND-type transporter AcrAB is involved in resistance toward apple phytoalexins and that it is required for successful colonization of the host plant [7]. Mutation of *acrB* in *E. amylovora* dramatically reduced tolerance to apple phytoalexins phloretin, naringenin, quercetin, and (+)-catechin. Herein, we report that the RND-type efflux transporters MdtABC and MdtUVW are also involved in secretion of antibacterial plant polyphenols, such as flavonoids and tannin. Furthermore, inactivation of the RND-type pumps MdtABC and MdtUVW, respectively, resulted in a reduced ability of *E. amylovora* to survive and multiply within apple rootstock MM106 (Figure 3). The population size of the *mdtABC* mutant was 3-fold lower than the population size of the wild type seven days after infection. The *mdtUVW* mutant showed an even greater reduction in its ability to multiply within apple rootstock reaching a 10-fold lower population size. In several bacterial pathosystems, virulence factors are induced by plant-derived signals. Expression analysis by qRT-PCR revealed an about 3-fold induction of the *mdtU* gene in the early infection phase from day 1 to 3 after infection of apple tissue with *E. amylovora* (Table 4). In contrast, the *mdtA* gene showed an about 50-fold increase in transcript abundance during growth of *E. amylovora* in apple rootstock. We identified tannin as an inducer of *mdtA* expression and as substrate of the MdtABC transporter (Tables 1 and 2). Condensed tannins, also called proanthocyanidins, are polymers formed by the condensation of flavonoids [40]. It has previously been demonstrated that the multidrug transporter MdtABC from *E. coli* was up-regulated in the presence of *Acacia mearnsii* tannin extract [20]. In addition to MdtABC, condensed tannins induced also other members of the envelope stress response regulated by the BaeSR two-component system in *E. coli*[20]. Indeed, the *baeSR* operon was also found to be up-regulated (2.8-fold) in *E. amylovora* during growth in medium containing tannin (data not shown). The response regulator BaeR was shown to positively regulate the expression of the *mdtABCD* locus in *E. coli* and *S. enterica*, leading to increased resistance to novobiocin, deoxycholate, SDS and β-lactam antibiotics [14,15,26,41].

The response regulator BaeR was shown to bind to the promoter region of the *mdtABCD* locus in *E. coli* and *S. enterica*[14,15]. In this study, we investigated whether BaeR is able to bind to the promoter regions of *mdtABC* and *mdtUVW* in *E. amylovora*. Our results show that BaeR binds to the promoter region of *mdtABC* but not to the *mdtUVW* promoter (Figure 2). Furthermore, overexpression of BaeR induced the expression of the RND-type efflux pumps a*crD* and *mdtABC* (Table 3).

Another envelope stress response, the Cpx two-component system, has been associated with the regulation of the MdtABC transporter in *E. coli*[27]. In order to analyze whether CpxR is involved in the expression of the RND-type pumps MdtABC and MdtUVW in *E. amylovora* Ea1189, we tested whether CpxR is able to bind to the promoter regions of the transporter genes (Figure 2). Our data suggest that CpxR does not directly interact with the promoter regions of *mdtABC* and *mdtUVW*. Moreover, overexpression of CpxR did not induce the expression of the RND pumps MdtABC and MdtUVW (Table 3).

We could demonstrate that flavonoids are natural substrates of the MdtABC and MdtUVW transporter from *E. amylovora*. The expression of these transporters was induced during infection of apple rootstock. Condensed tannins (polymers of flavanol units found in virtually all parts of a plant) induced the expression of the two-component system BaeSR which directly regulates the expression of the MdtABC pump in *E. amylovora*. Several flavonoids have been reported to possess antibacterial activities [42,43]. Their direct antibacterial activity may be attributable to different mechanisms including inhibition of nucleic acid synthesis, damage of cytoplasmic membrane function, inhibition of energy metabolism, inhibition of cell wall synthesis and inhibition of membrane synthesis [43,44]. Flavonoids cause stresses to the bacterial envelope. Therefore, it is not surprising that tannins induce the envelope stress response BaeSR in *E. amylovora*.

## Conclusions

The aim of the present study was the characterization of the RND-type multidrug efflux pumps MdtABC and MdtUVW from the plant pathogen *E. amylovora,* causing fire blight disease of apple, pear, and other members of the Rosaceae family*.* Our results suggest that MdtABC and MdtUVW play a role in survival and multiplication of *E. amylovora* in apple rootstocks as well as in the cell envelope stress response of the plant pathogen. The expression of both operons encoding these RND pumps was up-regulated *in planta*. We could identify the plant polyphenol tannin as inducer of *mdtABC* expression. The *mdtABC*- and *mdtUVW*-deficient mutants reached lower population sizes than the wild type in apple rootstock suggesting a role of the efflux pumps in resistance towards antimicrobial plant compounds, such as flavonoids. Moreover, we were able to demonstrate that the expression of *mdtABC* is activated by the two-component system BaeSR which is involved in the regulation of cell envelope stress responses.

## Methods

### Bacterial strains, plasmids and growth conditions

Bacterial strains used in the study are listed in Table 5 and plasmids in Additional file 4. *E. amylovora* strains were cultured at 28°C in Lysogeny Broth (LB) or double Yeast Trypton (dYT). *E. coli* XL-1 Blue was used as cloning host. DH5α λ-pir was used as host for the replacement vectors. BL21(DE3) was used as host for protein overexpression experiments. *E. coli* cells were routinely maintained at 37°C in dYT medium. Cultures harboring individual vectors were supplemented with 50 μg/ml ampicillin (Ap) for *E. coli* or 250 μg/ml for *E. amylovora*, 25 μg/ml chloramphenicol (Cm), 2 μg/ml gentamicin (Gm) or 25 μg/ml kanamycin (Km) when necessary. Bacterial growth was monitored using a spectrophotometer at 600 nm (OD_600_).

**Table 5:** Bacterial strains used in this study

**Strain**	**Relevant characteristics or genotype**^**a**^	**Reference or source**
*Escherichia coli*		
XL1-Blue	*recA*1 *endA*1 *gyrA*96 *thi-1 hsdR*17(r_K_^−^ m_K_^+^) *supE*44 *relA*1 *lac* [F’ *proAB lacI*^q^ ZΔM15Tn*10*(Tc^r^)]	Stratagene
BL21(DE3)	F^−^*ompT gal dcm lon hsdS*_B_(r_B_^−^ m_B_^−^) λ(DE3)	Novagen
S17-1	Tc^r^ Sm^r^, *recA pro hsdR* (RP4-2-Tc::Mu-Km::Tn*7*)	[45]
S17-1 λ-pir	λpir phage lysogen of S17-1	[45]
DH5α λ-pir	F^−^*supE*44 Δ*lac*U169 (Φ*lacZ*ΔM15) *recA*1 *endA*1 *hsdR*17(r_K_^−^ m_K_^+^) *thi-1 gyrA*96 *relA*1, λpir phage lysogen	D. Lies, Caltech
*Erwinia amylovora*		
Ea1189	wild type	GSPB^*b*^
Ea1189-3	Ea1189, Km^r^ cassette in *acrB*	[7]
Ea1189.ΔmdtABC	Ea1189, *mdtABC* deletion mutant	This study
Ea1189.ΔmdtUVW	Ea1189, *mdtUVW* deletion mutant	This study

^*a*^Antibiotic resistance: Ap^r^, ampicillin; Cm^r^, chloramphenicol; Km^r^, kanamycin; Sm^r^, streptomycin; Tc^r^, tetracycline.

^*b*^GSPB, Göttinger Sammlung Phytopathogener Bakterien, Göttingen, Germany.

### PCR amplifications, modifications and protein purification

PCR primers are listed in Additional file 9. Primers were designed based on the genome sequence of *E. amylovora* CFBP1430 available from NCBI (NC_013961.1). Screening PCR reactions were carried out using DreamTaq DNA polymerase (Thermo Scientific) in accordance with the manufacturer’s instructions. Annealing temperatures were optimized based on the melting temperatures of the respective primers. For high fidelity PCR reactions, Phusion DNA polymerase (Thermo Scientific) or Q5 DNA polymerase (NEB) was used.

Restriction enzyme (Thermo Scientific) and T4 DNA ligase (Thermo Scientific) reactions were performed following the manufacturer’s instructions at the appropriate temperature where all ligation reactions were incubated at room temperature.

DNA purifications were either performed using the GeneJET PCR purification kit (Thermo Scientific) or the GeneJET Gel extraction kit (Thermo Scientific) according to the manufacturer’s instructions.

### Construction of *mdtABC*- and *mdtUVW*-deficient mutants of *E. amylovora*

The construction of the knockout vectors was based on the protocol described by Zumaquero et al. [46]. Briefly, approximately 500 to 700 bp flanking the 5’ and 3’ regions of the ORF’s to be deleted were PCR-amplified using primer pairs mdtABC-A1 and mdtABC-A2, mdtABC-B1 and mdtABC-B2, mdtUVW-A1 and mdtUVW-A2, mdtUVW-B1 and mdtUVW-B2. Primers A2 and B1 share a 20-nucleotide homologous sequence at their 5’ ends, consisting of the T7 primer sequence and a *Kpn*I restriction site [46]. After amplification, the obtained fragments were gel-purified and approximately 40 ng of an A and B fragment were used for a fusion PCR reaction with primers A1 and B2. The resulting fusion product was gel-purified, cloned into pJET1.2 and confirmed by sequencing. Next, a chloramphenicol cassette, flanked by Flp-*FRT* sites, was cut from plasmid pFCm1 and subsequently inserted into the *Kpn*I-digested pJET constructs. The deletion alleles were cut with *Eco*RI and further ligated into *Eco*RI-digested pCAM-Km, yielding the final replacement plasmids pCAM-Km.mdtABC-Cm and pCAM-Km.mdtUVW-Cm. The plasmids were transformed into electrocompetent cells of *E. amylovora* Ea1189, which subsequently were grown for 3 h at 28°C in dYT broth. Putative mutants were screened for homologous recombination events by testing their antibiotic resistance. Mutants that resulted from single crossover events were identified by their ability to grow on plates containing Km. In order to confirm gene deletion through a double crossover event in Cm-resistant and Km-sensitive colonies, primers which bind upstream and downstream of the A and B fragment used for generation of the gene replacement vector were designed. PCRs were done using these locus-specific primers with outward-facing primers binding within the Cm cassette. Amplified PCR products were verified by sequencing.

The Cm-*FRT* cassette was finally excised using the temperature-sensitive plasmid pCP20 that carries the yeast Flp recombinase gene [47,48]. Briefly, Cm-resistant mutants of Ea1189 were transformed with pCP20 and selected at 28°C on LB plates containing Ap. Subsequently, Ap-resistant transformants were streaked on non-selective agar plates and incubated at 43°C for 1 h, following incubation at 28°C for 48 to 60 h. Single colonies were selected and tested on agar plates containing Cm or Ap to confirm successful excision of the Cm cassette and loss of plasmid pCP20.

### Cloning of the *mdtABC* and *mdtUVW* regions of *E. amylovora*

The *mdtABC* and *mdtUVW* operons, including their promoter regions, were PCR-amplified using the primer pairs mdtABC_P_SacI/mdtABC_ApaI (7702 bp) and mdtUVW_P_SacI/mdtUVW_ApaI (7810 bp), respectively. The obtained PCR products were sequenced and subsequently cloned into *Sac*I-*Apa*I-digested pBlueScript II KS(+) to obtain expression from the native promoters (pBlueKS.mdtABC-ext, pBlueKS.mdtUVW-ext) or into pBlueScript II SK(+) to obtain expression from the native promoters and P_*lacZ*_ (pBlueSK.mdtABC-ext, pBlueSK.mdtUVW-ext).

In order to study the effect of β-lactam antibiotics on growth of *E. amylovora* containing *mdtABC* and *mdtUVW* overexpression plasmids, the *mdtABC* and *mdtUVW* regions were cloned into pBlueScript II KS vectors where the Ap^r^ gene was exchanged by a Sm^r^ gene (Additional file 4).

### Drug susceptibility tests

The minimal inhibitory concentrations (MICs) of drugs for *E. amylovora* strains were determined by a 2-fold dilution assay in a 96-well plate using Mueller-Hinton broth (MHB). All tests were performed at least in triplicate following the Clinical and Laboratory Standards Institute recommendations [49]. Growth of bacteria at 28°C was examined by visual inspection after 48 h incubation. The MIC was defined as the lowest concentration of an antibiotic that completely prevented visible cell growth.

### Generation of promoter-*egfp* fusion constructs

Transcriptional fusions between the promoter regions of *mdtABC* and *mdtUVW*, respectively, and *egfp* were created using a previously described PCR-based method [50]. Briefly, a 294-bp fragment containing the upstream region of *mdtABC* was amplified using the primer mdtABC_up and the reverse primer mdtABC-P-egfp containing a 24-nt extension homologous to the start of the *egfp* gene. The *mdtUVW* upstream region (266 bp) was amplified using the primer mdtUVW_up and the reverse primer mdtUVW-P-egfp. Next, a 916-bp fragment containing the reporter gene *egfp* was amplified using the primer pair egfp-ATG and egfp-Cm and the plasmid pBBR.egfp.TIR as template [7]. All PCR products were gel-purified and validated by sequencing. For the fusion reaction, 60 ng of a PCR fragment containing a promoter region were mixed with 20 ng of the reporter gene fragment. For fusion of the *mdtABC* promoter to the *egfp* gene, the primers mdtABC-P_SacII and uidA-t0-KpnI were used. The primers mdtUVW-P_SacII and uidA-t0-KpnI were used in a PCR to fuse the *mdtUVW* promoter to *egfp*. The fusion products were gel-purified and cloned into *Sac*II-*Kpn*I-treated pBBR1MCS, in opposite direction to the *lacZ* promoter, yielding plasmids pBBR.mdtABC-Pro.egfp and pBBR.mdtUVW-Pro.egfp.

### Promoter activity of *mdtABC* and *mdtUVW in vitro*

The reporter gene *egfp* was employed to study the impact of diverse antimicrobial substances on promoter activities of *mdtABC* and *mdtUVW* in *E. amylovora*. Plasmids carrying the transcriptional fusions were transformed into Ea1189. Antimicrobial compounds were added to the bacterial cells in 96-well microtiter plates by the 2-fold dilution method as described for MIC assays. EGFP fluorescence of the cells following exposure to various concentrations of the substrates was measured 48 hours after incubation at 28°C using the microplate reader Infinite M1000 PRO (Tecan, Crailsheim, Germany) with an excitation wavelength of 470 nm and emission detection at 516 nm.

Fluorescence values obtained were plotted versus optical densities in a scatter plot. A best-fit linear regression line was added to the plot and a 95% confidence interval determined using the software package for the R statistical language and environment [51]. Data points that did not meet the confidence interval criteria indicate fluorescence values higher than the average, thereby suggesting promoter induction by the respective compound. The following substrates were applied to this assay: (+)-catechin, acridine orange, acriflavine, amikacin, azithromycin, benzalkonium chloride, berberine, bile salts, cadmium acetate, chloramphenicol, ciprofloxacin, clarithromycin, clotrimazole, cobalt chloride, copper sulfate, crystal violet, daidzein, deoxycholate, erythromycin, ethidium bromide, fusaric acid, fusidic acid, genistein, gentamicin, josamycin, luteolin, myricetin, naladixic acid, naringenin, nickel chloride, nitrofurantoin, norfloxacin, novobiocin, orobol, phloretin, polymyxin B, quercitin, rhodamine 6G, rifampicin, roxithromycin, SDS, silver nitrate, sodium arsenate, sodium tungstate, streptomycin, tetracycline, tetraphenylphosphonium chloride, tobramycin and zinc sulfate.

### Plant material and inoculation procedures

Apple plants (rootstock Malus MM106) were grown in a greenhouse at 20 to 25°C, 60% humidity, and 12 h photoperiod (15,000 lx). *E. amylovora* strains grown on LB agar for 24 h, were resuspended and diluted to an OD_600_ of 1.0 in sterile demineralized water. Apple plants were inoculated by prick technique [52]. Each bacterial strain was inoculated into one shoot of minimum five single plants. A bacterial suspension (5 μl) was placed onto each wound on the shoot tip. Plants were monitored for symptom development daily. Survival of bacteria in plant tissue was examined by re-isolation of bacterial cells 1, 3 and 7 day(s) after inoculation, respectively, from 1 cm of the shoot tip around the inoculation area. The experiment was repeated at least three times.

In order to analyze the abundance of *mdtA* and *mdtU* mRNA transcripts in *E. amylovora* Ea1189 during growth in apple rootstock MM106, total RNA was isolated from infected apple shoots 1, 3 and 7 day(s) post inoculation, respectively. Five individual wounds were pooled together, homogenized in sterile water and centrifuged for 2 min at 4000 rpm. The supernatant was transferred to 15 ml killing buffer (20 mM Tris–HCl, pH 7.5; 20 mM NaN_3_) [53] and centrifuged for 20 min at 4000 rpm. The supernatant was decanted and the pellet frozen at-80°C for further RNA extraction.

### Virulence assay on immature pears

Virulence of *E. amylovora* Ea1189 and the *mdtABC*, *mdtUVW*, *baeR* and *cpxR* mutants was determined on immature pears (*Pyrus communis* L. cv. ‘Bartlett’). Bacteria, grown on LB agar plates at 28°C for 24 h, were resuspended and adjusted to an OD_600_ of 1.0 in sterile demineralized water for inoculation. Immature pear fruits were surface-sterilized and pricked with a sterile needle as described previously [54]. Wounds were inoculated with 5 × 10^6^ CFU/ml and incubated in a humidified chamber at 18°C and 28°C, respectively for 6 to 14 days. Disease symptoms were visually recorded by means of necrosis surrounding the infection site. Five fruits were assayed and the experiment was repeated twice.

To analyze gene expression of *E. amylovora* Ea1189 during growth on pear fruits, immature fruits were cut in slices (approx. 0.5 cm). Five slices were inoculated with 100 μl of a bacterial suspension adjusted to an OD_600_ of 1.0 in sterile demineralized water. The suspension was evenly distributed on the slice and incubated for 12 hours in a humidified chamber at room temperature. Next, the upper layer of the surface was scratched from the five slices, resuspended in 25 ml of PBS and centrifuged for 2 min at 4000 rpm. The supernatant was transferred to 15 ml killing buffer and further processed as described above.

### RNA isolation and quantitative RT-PCR

Cell cultures were grown in LB broth until the desired optical densities was reached. An aliquot containing 15 × 10^9^ CFU (equivalent of 15 ml culture with an OD_600_ of 1.0) was transferred to 15 ml killing buffer and centrifuged for 20 min at 4000 rpm. The supernatant was decanted and the pellet frozen at-80°C for further RNA extraction.

Total RNA was isolated by acid phenol/chloroform extraction [53]. The obtained RNA was DNAse (Ambion/Life Technologies) treated and subsequently checked for purity by gel electrophoresis and determination of the A_260_/A_280_ and A_260_/A_230_ ratios using a Nanodrop ND-2000 spectrophotometer (Thermo Fischer Scientific). High quality RNA was reverse transcribed and amplified with the OneStep RT-PCR Kit according to the manufacturer’s protocol (Qiagen). Template RNA (5 ng) was used in a standard 25-μl qRT-PCR reaction with specific primers (Additional file 5). As control, RNA samples without reverse transcriptase were included to detect possible DNA contaminations.

For analysis, a Mastercycler ep *realplex*^2^ gradient S (Eppendorf, Hamburg, Germany) was used. Cycling parameters included a 15 min initial denaturation at 95°C to activate the DNA polymerase followed by 40 cycles consisting of 15 sec at 95°C, 30 sec at 55°C and 30 sec at 72°C. The final step consisted of 1 min at 95°C and 30 sec at 55°C. A melting curve analysis with a temperature ramp from 25°C to 95°C in 20 min was performed at the end of each run to determine specificity of amplified qPCR products.

Each sample was analyzed for gene expression in triplicate. Quantification of mRNA transcripts was performed by the comparative C_t_ method. Briefly, the C_t_ values of the samples of interest were compared with a non-treated sample. All C_t_ values were normalized to the housekeeping gene *recA*, which shows constant expression at different ODs and medium compositions as well as similar amplification efficiency to the target genes [34,55]. The comparative C_t_ method was calculated by $2^{-\left(\Delta C_{t},\mathrm{sample}-\Delta C_{t},\mathrm{reference}\right)}$, where ΔC_t_ was normalized to the endogenous housekeeping gene *recA*. Subsequently, fold-changes between the samples were determined based on the calculated C_t_ method.

### Purification of the BaeR and CpxR protein

Protein purification was carried out using the Ni-NTA Spin Kit (Qiagen) following the manufacturer’s instructions. Briefly, *E. coli* BL21(DE3) harboring the expression plasmid pET28a.baeR or pET28a.cpxR was grown at 37°C in 50 ml LB broth supplemented with kanamycin. When the culture reached an OD_600_ of 0.6, IPTG was added to a final concentration of 1 mM and the culture further incubated for 4 hours. Cells were harvested by centrifugation at 4000 rpm for 20 min. The obtained cell pellet was lysed using lysozyme (1 mg/ml) for 1 hour on ice and then purified on the Ni-NTA spin columns. Lysate, wash fractions and eluted protein samples were collected and further analyzed by SDS-PAGE.

### Electrophoretic mobility shift assay

DNA fragments used for the electrophoretic mobility shift assay (EMSA) were PCR amplified using Cy5-labeled primers to perform a non-radioactive EMSA. DNA fragments used were the upstream region of *mdtABC* (290 bp), *mdtUVW* (276 bp) and as control, a fragment of the *tolC* gene (248 bp). Approximately 0.3 pmol of Cy5-labeled DNA was mixed with increasing concentrations of His-tagged BaeR or CpxR protein in a binding buffer reaction (10 mM Tris–HCl, pH 7.5; 50 mM KCl; 5 mM MgCl_2_; 1 mM DTT; 2.5% glycerol). To decrease unspecific binding, 50 ng competitor DNA (Salmon sperm DNA, AppliChem) was added to the reaction. Incubation was done at room temperature for 30 min. The total reaction was run on a native 4% polyacrylamide gel in 0.5× Tris-borate-EDTA (TBE) buffer at constant 25 mA. After electrophoresis, fluorescence signals of the labeled DNA were visualized using a FLA-3000 phosphorimager (Raytest, Straubenhardt, Germany).

### Overexpression of the BaeR and CpxR

To investigate whether overexpression of the response regulators BaeR and CpxR affects expression of the multidrug efflux systems *mdtABC* and *mdtUVW*, *baeR* and *cpxR* were cloned into the pBAD24 vector where gene expression is under control of the arabinose-inducible promoter P_BAD_. Hence, *baeR* was amplified using the primer pairs baeR_SacII/baeR_ApaI and *cpxR* using cpxR(HindIII)_fwd/cpxR(EcoRI)_rev. The obtained PCR products were sequenced and subsequently cloned into *Sac*II-*Apa*I-digested pBAD24 (pBAD24.baeR) or *Hind*III/*EcoR*I-digested pBAD24 (pBAD24.cpxR). Next, plasmids were transformed into *E. amylovora* Ea1189 and protein expression was induced by adding 1% L-arabinose when cultures reached an OD_600_ of 0.5. Cells were further incubated for 1 hour at 28°C and subsequently transferred into killing buffer. Afterwards, cells were harvested by centrifugation for 20 min at 4000 rpm. The supernatant was decanted and the pellet frozen at-80°C for further RNA extraction.

## Competing interests

The authors declared that they have no competing interests.

## Authors’ contributions

DP carried out the molecular work, participated in the bioinformatical analysis and drafted the manuscript. HW conceived of the study, participated in its design and coordination and helped to draft the manuscript. All authors read and approved the final manuscript.

## Supplementary Material

Additional file 1 **BLASTP results for MdtABC and MdtUVW from *E. amylovora *** **Ea1189 and MdtABC from *E. coli *** **W3110.** Click here for file

Additional file 2 **Modified view of the genomic organization of the *mdtABCD *** **locus from *E. coli *** **MG1655 and the *mdtABC *** **locus from *E. amylovora *** **CFBP1430.** Visualization was obtained by the Artemis Comparison Tool [56]. The dark areas indicate homologous regions with a minimum identity cutoff score of 50% and a maximum identity cutoff score of 89%. The alignment was performed using the nucleotide search BLASTN from NCBI. Click here for file

Additional file 3 **Transmembrane protein topology of (A) MdtABC from *E. amylovora *** **Ea1189, (B) MdtUVW from *E. amylovora *** **Ea1189 and (C) MdtABC from *E. coli *** **W3110.** The upper line indicates the predicted topology from TOPCONS [31] based on amino acid sequences. Red lines indicate an inner membrane orientation; blue lines indicate an outer membrane orientation. Grey boxes indicate transmembrane helices spanning from the inside to the outside, white boxes indicate transmembrane helices spanning from the outside to the inside. Below the line is a graphical interpretation of the reliability of the prediction for each amino acid. Click here for file

Additional file 4 Plasmids used in this study. Click here for file

Additional file 5 **Promoter activities of *mdtABC *** **and *mdtUVW *** **from *E. amylovora *** **Ea1189 determined in the course of growth.** (A) Relative mRNA transcript abundance of *mdtABC* and *mdtUVW* during cellular growth of Ea1189 as determined by quantitative RT-PCR. The relative mRNA level was related to the highest mean value determined for a gene, which was defined as 100%. (B) Expression of *mdtABC* and *mdtUVW* as determined by transcriptional fusions with the reporter gene *egfp. E. amylovora* wild type was transformed with pBBR.mdtABC-Pro.egfp and pBBR.UVW-Pro.egfp, respectively. To assay fluorescence of the enhanced green fluorescent protein during growth of cells in LB broth, aliquots were harvested at distinct optical densities and adjusted to an OD_600_ value of 0.1. Experiments were performed in triplicates with similar results. OD_600_, optical density at 600 nm. Click here for file

Additional file 6 **Sequence alignment of the amino acid sequences of (A) BaeR from *E. amylovora *** **Ea1189 and *E. coli*** **W3110 (YP_490321.1) and (B) CpxR from** ***E. amylovora *** **Ea1189 and *E. coli*** **W3110 (YP_491538.1).** Analysis was performed with Clustal Omega and Jalview [28,57]. BaeR of Ea1189 is 74% identical to BaeR of *E. coli*. CpxR of Ea1189 is 90% identical to CpxR of *E. coli*. Identical amino acid residues are shown in blue. Yellow bars show a quantitative measurement of conserved physico-chemical properties where the highest score shows amino acids of the same physico-chemical class. Black bars indicate predicted response regulator receiver domains and grey bars indicate predicted transcriptional regulatory domains from *E. amylovora* Ea1189 as determined by using PFAM [58]. Click here for file

Additional file 7 Virulence assay on apple rootstock MM106. Click here for file

Additional file 8 **Symptoms of *E. amylovora *** **Ea1189 and *acrB, *** **Δ** ***mdtABC *** **and Δ** ***mdtUVW *** **mutants in immature pear at (A) 18°C, 14 days post inoculation and (B) 28°C, 6 days post inoculation.** Pictures represent one out of ten pear fruits per infection. Click here for file

Additional file 9 Primers used in this study. Click here for file
