# Supplementary material for: Characterization and regulation of the Resistance-Nodulation-Cell Division-type multidrug efflux pumps MdtABC and MdtUVW from the fire blight pathogen Erwinia amylovora
Source: BMC Microbiol. 2014 Jul 11;14:185. doi: 10.1186/1471-2180-14-185 (PMC4107485; doi:10.1186/1471-2180-14-185)
Supplement: Additional file 1 — BLASTP results for MdtABC and MdtUVW from E. amylovora Ea1189 and MdtABC from E. coli W3110. [file 1471-2180-14-185-S1.pdf]

Additional File 1. BLASTP results for MdtABC and MdtUVW from *E. amylovora* Ea1189 and MdtABC from *E. coli* W3110 (Ec).[a]

	**MdtA**	**MdtU**	**MdtA Ec**
Accession number	YP_003531620.1	YP_003530041.1	YP_490316.1
Length (aa)	405	421	415
**MdtA**	100%	46%	**65%**
**MdtU**	49%	100%	46%
**MdtA Ec**	**65%**	44%	100%

	**MdtB**	**MdtV**	**MdtB Ec**
Accession number	YP_003531621.1	YP_003530042.1	YP_490317.1
Length (aa)	1039	1074	1040
**MdtB**	100%	62%	**81%**
**MdtV**	59%	100%	61%
**MdtW**	48%	44%	47%
**MdtB Ec**	**82%**	60%	100%

	**MdtC**	**MdtW**	**MdtC Ec**
Accession number	YP_003531622.1	YP_003530043.1	YP_490318.1
Length (aa)	1024	1032	1025
**MdtC**	100%	54%	**74%**
**MdtV**	46%	45%	47%
**MdtW**	54%	100%	56%
**MdtC Ec**	**74%**	56%	100%

[a] Bold numbers indicate the highest values of sequence identity.
