# Supplementary figures and images for: Characterization and regulation of the Resistance-Nodulation-Cell Division-type multidrug efflux pumps MdtABC and MdtUVW from the fire blight pathogen Erwinia amylovora
Source: BMC Microbiol. 2014 Jul 11;14:185. doi: 10.1186/1471-2180-14-185 (PMC4107485; doi:10.1186/1471-2180-14-185)

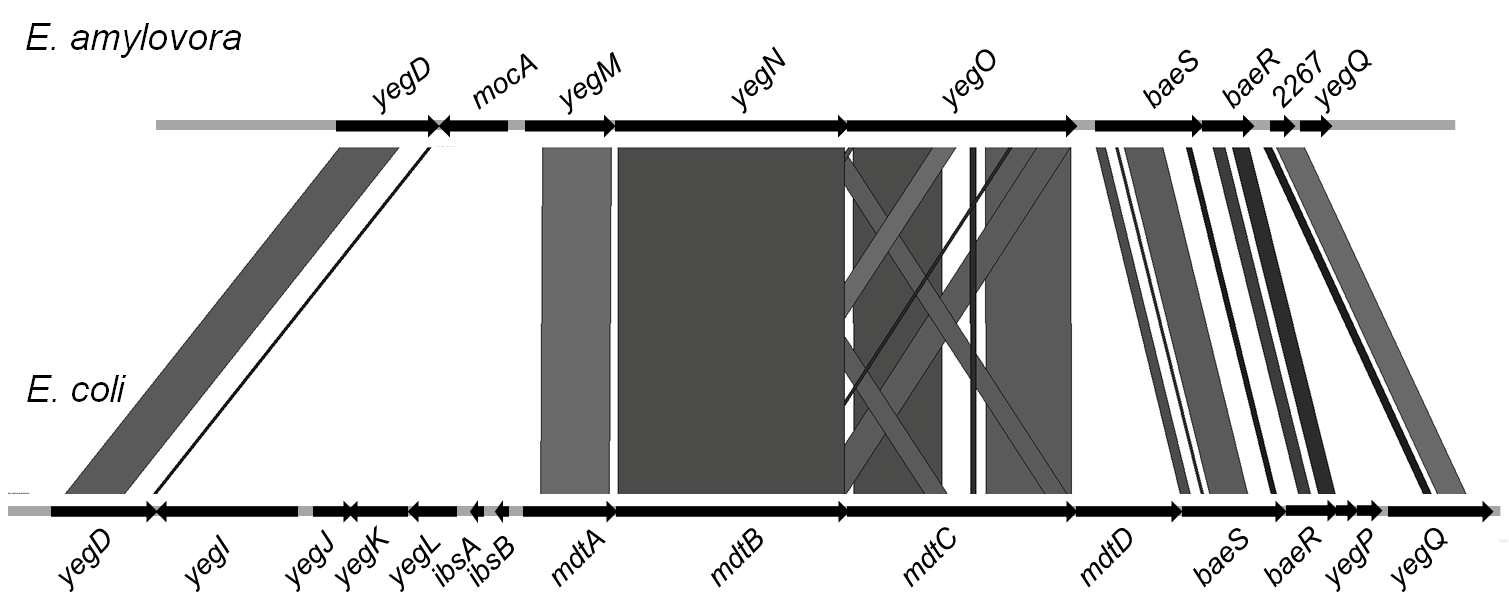

Supplement: Additional file 2 — Modified view of the genomic organization of the mdtABCD locus from E. coli MG1655 and the mdtABC locus from E. amylovora CFBP1430. Visualization was obtained by the Artemis Comparison Tool [56]. The dark areas indicate homologous regions with a minimum identity cutoff score of 50% and a maximum identity cutoff score of 89%. The alignment was performed using the nucleotide search BLASTN from NCBI. [file 1471-2180-14-185-S2.tiff]

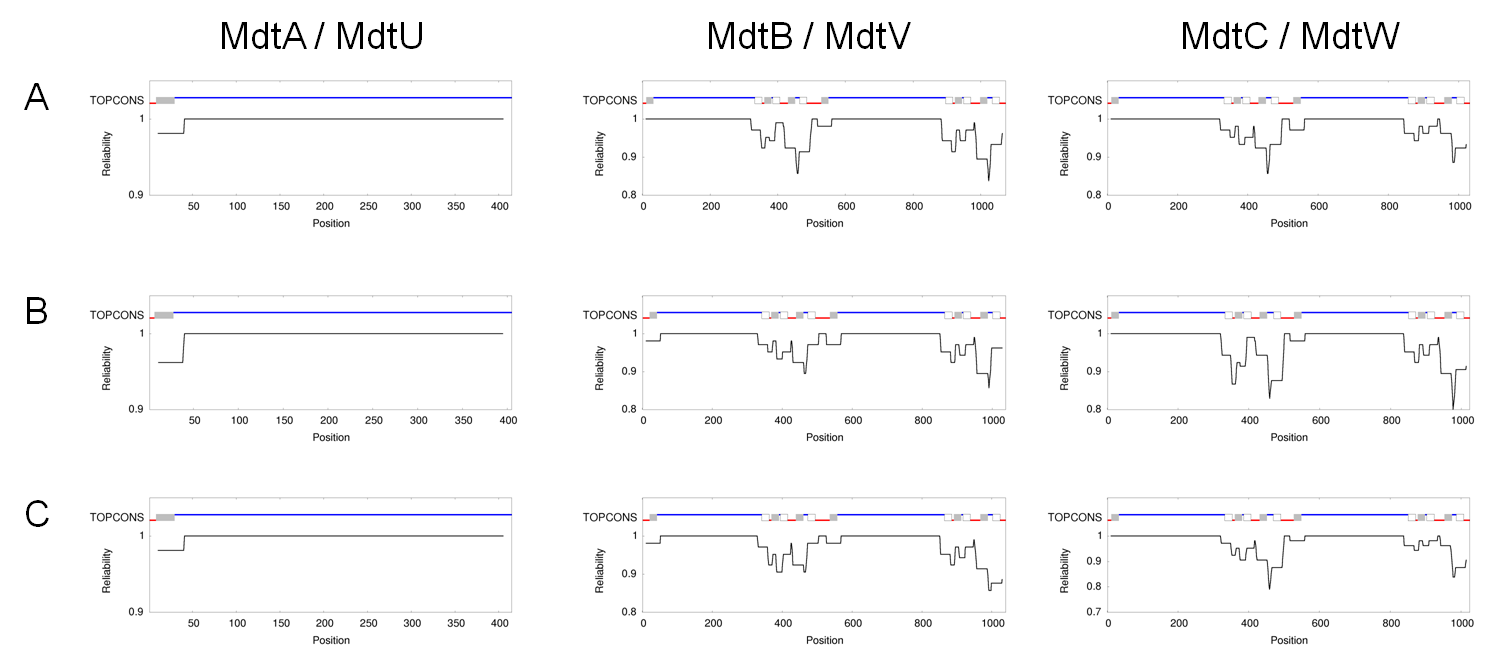

Supplement: Additional file 3 — Transmembrane protein topology of (A) MdtABC from E. amylovora Ea1189, (B) MdtUVW from E. amylovora Ea1189 and (C) MdtABC from E. coli W3110. The upper line indicates the predicted topology from TOPCONS [31] based on amino acid sequences. Red lines indicate an inner membrane orientation; blue lines indicate an outer membrane orientation. Grey boxes indicate transmembrane helices spanning from the inside to the outside, white boxes indicate transmembrane helices spanning from the outside to the inside. Below the line is a graphical interpretation of the reliability of the prediction for each amino acid. [file 1471-2180-14-185-S3.tiff]

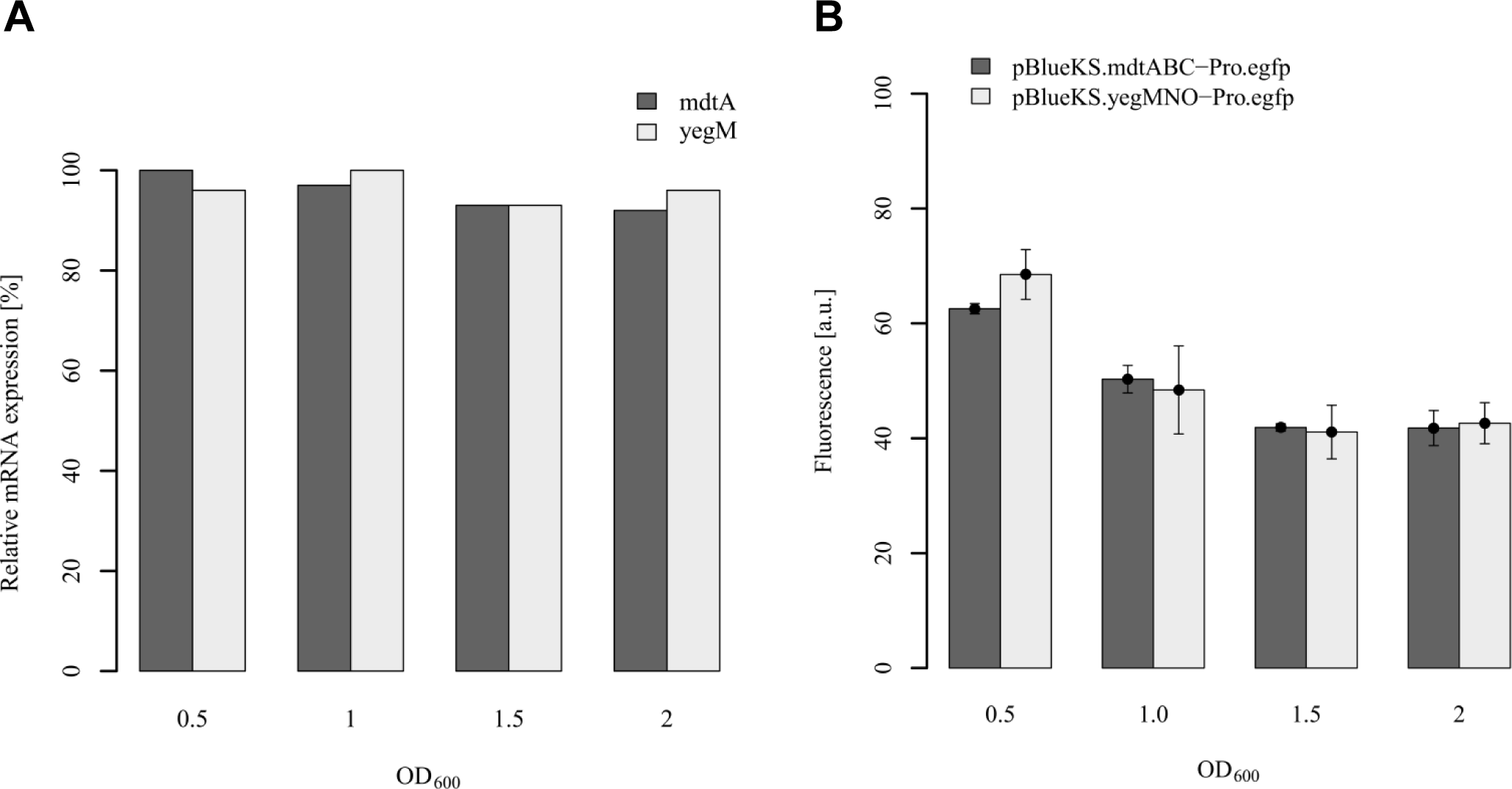

Supplement: Additional file 5 — Promoter activities of mdtABC and mdtUVW from E. amylovora Ea1189 determined in the course of growth. (A) Relative mRNA transcript abundance of mdtABC and mdtUVW during cellular growth of Ea1189 as determined by quantitative RT-PCR. The relative mRNA level was related to the highest mean value determined for a gene, which was defined as 100%. (B) Expression of mdtABC and mdtUVW as determined by transcriptional fusions with the reporter gene egfp. E. amylovora wild type was transformed with pBBR.mdtABC-Pro.egfp and pBBR.UVW-Pro.egfp, respectively. To assay fluorescence of the enhanced green fluorescent protein during growth of cells in LB broth, aliquots were harvested at distinct optical densities and adjusted to an OD600 value of 0.1. Experiments were performed in triplicates with similar results. OD600, optical density at 600 nm. [file 1471-2180-14-185-S5.tiff]

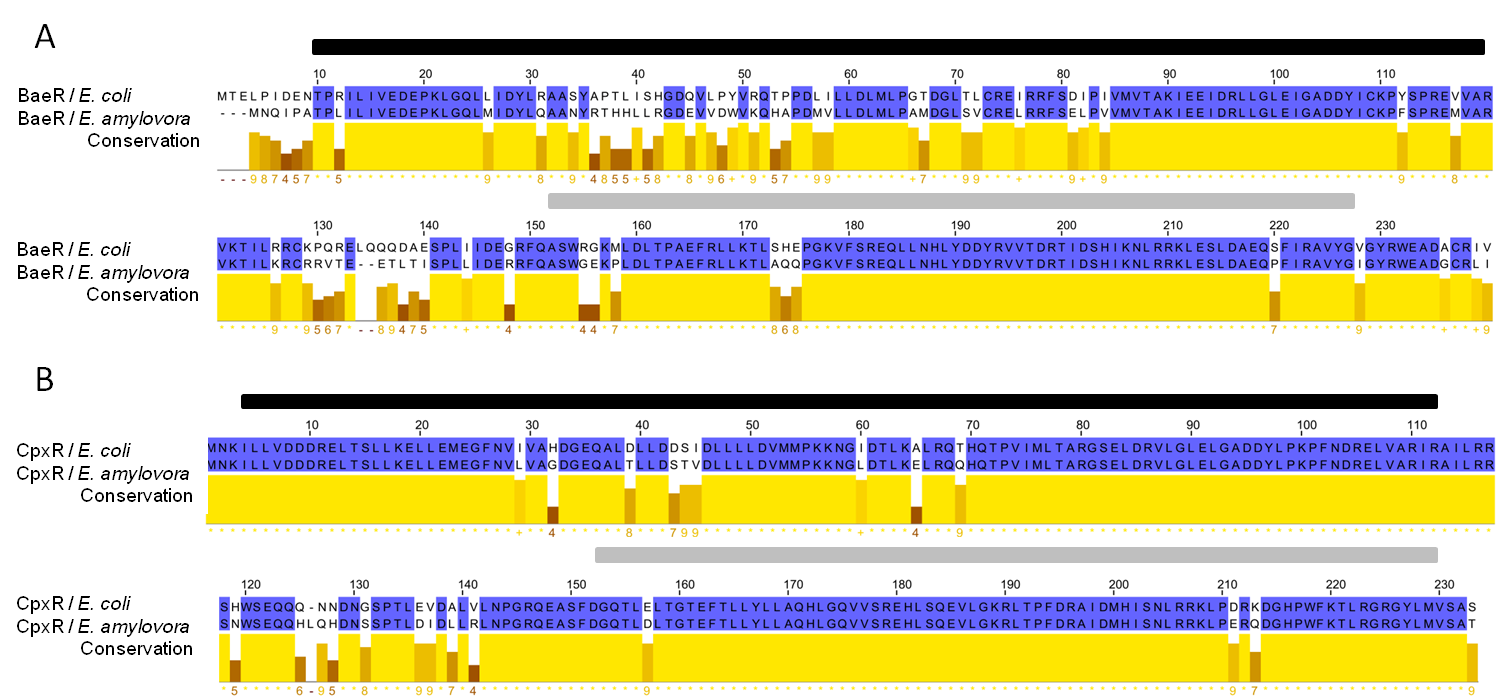

Supplement: Additional file 6 — Sequence alignment of the amino acid sequences of (A) BaeR from E. amylovora Ea1189 and E. coli W3110 (YP_490321.1) and (B) CpxR from E. amylovora Ea1189 and E. coli W3110 (YP_491538.1). Analysis was performed with Clustal Omega and Jalview [28,57]. BaeR of Ea1189 is 74% identical to BaeR of E. coli. CpxR of Ea1189 is 90% identical to CpxR of E. coli. Identical amino acid residues are shown in blue. Yellow bars show a quantitative measurement of conserved physico-chemical properties where the highest score shows amino acids of the same physico-chemical class. Black bars indicate predicted response regulator receiver domains and grey bars indicate predicted transcriptional regulatory domains from E. amylovora Ea1189 as determined by using PFAM [58]. [file 1471-2180-14-185-S6.tiff]

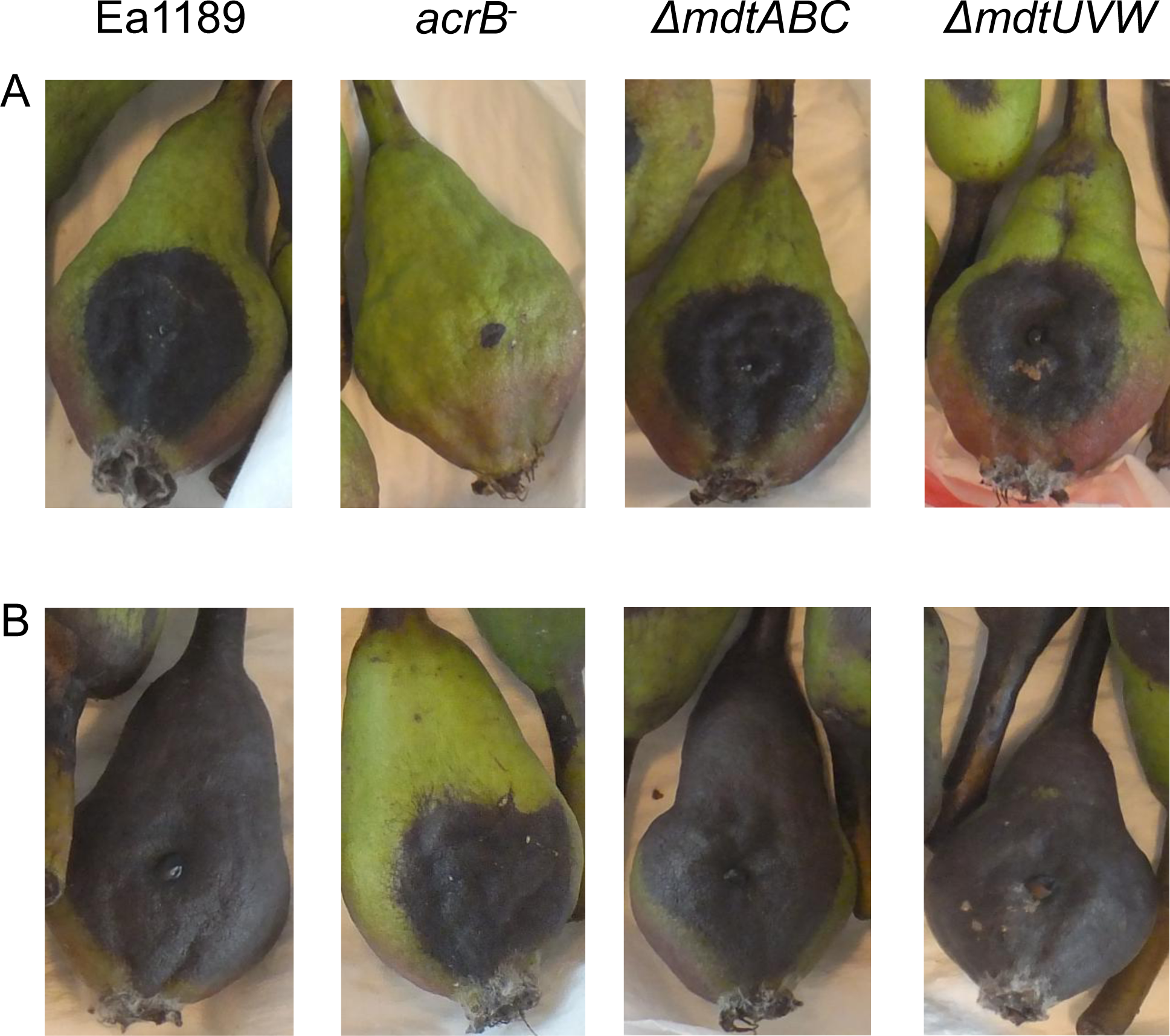

Supplement: Additional file 8 — Symptoms of E. amylovora Ea1189 and acrB, Δ mdtABC and Δ mdtUVW mutants in immature pear at (A) 18°C, 14 days post inoculation and (B) 28°C, 6 days post inoculation. Pictures represent one out of ten pear fruits per infection. [file 1471-2180-14-185-S8.tiff]
