# Supplementary material for: Characterization and regulation of the Resistance-Nodulation-Cell Division-type multidrug efflux pumps MdtABC and MdtUVW from the fire blight pathogen Erwinia amylovora
Source: BMC Microbiol. 2014 Jul 11;14:185. doi: 10.1186/1471-2180-14-185 (PMC4107485; doi:10.1186/1471-2180-14-185)
Supplement: Additional file 4 — Plasmids used in this study. [file 1471-2180-14-185-S4.pdf]

Additional File 4. Plasmids used in this study.

Plasmid	Relevant characteristics[a]	Reference or source
pJET1.2	Ap^r, rep (pMB1) from pMBI responsible for replication	Thermo Scientific
pBlueScript II SK(+)	Ap^r, ColE1 origin	Stratagene
pBlueScript II KS(+)	Ap^r, ColE1 origin	Stratagene
pBBR1MCS	Cm^r, origin from pBBR1 from *Bordetella bronchiseptica*, broad-host-range cloning vector	[1]
pCAM-MCS	Ap^r, R6K origin, pCAM140-derivative without mini-Tn5, contains the MCS of pBluescript II SK (+)	[2]
pCAM-Km	Km^r, variant of the gene replacement vector pCAM-MCS, Ap^r replaced by Km^r	This study
pFCm1	Ap^r, Cm^r, source of Cm^r cassette flanked by FRT sequences	[3]
pCP20	Cm^r, Ap^r, contains yeast Flp recombinase gene, rep (pSC101) responsible for temperature-sensitive replication	[3]
pCAM-Km.mdtUVW-Cm	Km^r, Cm^r, contains a 1.3-kb fusion fragment of *mdtU* and *mdtW* from *E. amylovora* Ea1189, insertion of 1135-bp Cm-FRT cassette from pFCm1 in *Kpn*I site of the fusion fragment	This study
pCAM-Km.mdtABC-Cm	Km^r, Cm^r, contains a 1.2-kb fusion fragment of *mdtA* and *mdtC* from *E. amylovora* Ea1189, insertion of 1135-bp Cm-FRT cassette from pFCm1 in *Kpn*I site of the fusion fragment	This study
pBlueSK.mdtABC	Ap^r, contains a 7.4-kb fragment carrying *mdtABC* of *E. amylovora* Ea1189 under control of *lac* promoter	This study
pBlueSK.mdtABC-ext	Ap^r, contains a 7.7-kb fragment carrying *mdtABC* of *E. amylovora* Ea1189 including upstream promoter region under control of *lac* promoter	This study
pBlueKS.mdtABC	Ap^r, contains a 7.4-kb fragment carrying *mdtABC* of *E. amylovora* Ea1189 in opposite orientation with respect to *lac* promoter	This study
pBlueKS.mdtABC-ext	Ap^r, contains a 7.7-kb fragment carrying *mdtABC* of *E. amylovora* Ea1189 including upstream promoter region in opposite orientation with respect to *lac* promoter	This study
pBlueSK.mdtUVW	Ap^r, contains a 7.6-kb fragment carrying *mdtUVW* of *E. amylovora* Ea1189 under control of *lac* promoter	This study
pBlueSK.mdtUVW-ext	Ap^r, contains a 7.8-kb fragment carrying *mdtUVW* of *E. amylovora* Ea1189 including upstream promoter region under control of *lac* promoter	This study
pBlueKS.mdtUVW	Ap^r, contains a 7.6-kb fragment carrying *mdtUVW* of *E. amylovora* Ea1189 in opposite orientation with respect to *lac* promoter	This study
pBlueKS.mdtUVW-ext	Ap^r, contains a 7.8-kb fragment carrying *mdtUVW* of *E. amylovora* Ea1189 including upstream promoter region in opposite orientation with respect to *lac* promoter	This study
pBlueKS-SM	Sm^r, variant of pBlueScript II KS(+), Ap^r replaced by Sm^r in *Dra*I site of pBlueScript II KS(+)	This study
pBlueSK-SM	Sm^r, variant of pBlueScript II SK(+), Ap^r replaced by Sm^r in *Dra*I site of pBlueScript II SK(+)	This study
pBlueSK-SM.mdtABC	Sm^r, variant of pBlueSK.mdtABC	This study
pBlueSK-SM.mdtABC-ext	Sm^r, variant of pBlueSK.mdtABC-ext	This study
pBlueKS-SM.mdtABC	Sm^r, variant of pBlueKS.mdtABC	This study
pBlueKS-SM.mdtABC-ext	Sm^r, variant of pBlueKS.mdtABC-ext	This study
pBlueSK-SM.mdtUVW	Sm^r, variant of pBlueSK.mdtUVW	This study
pBlueSK-SM.mdtUVW -ext	Sm^r, variant of pBlueSK.mdtUVW-ext	This study
pBlueKS-SM.mdtUVW	Sm^r, variant of pBlueKS.mdtUVW	This study
pBlueKS-SM.mdtUVW -ext	Sm^r, variant of pBlueKS.mdtUVW-ext	This study
pBlueKS.baeR	Ap^r, contains a 0.7-kp fragment carrying *baeR* of *E. amylovora* Ea1189	This study
pBlueKS.baeR-ext	Ap^r, contains a 1.0-kp fragment carrying *baeR* of *E. amylovora* Ea1189 including upstream promoter region	This study
pBlueSK.baeR	Ap^r, contains a 0.7-kp fragment carrying *baeR* of *E. amylovora* Ea1189 under control of *lac* promoter	This study
pBlueSK.cpxR	Ap^r, contains a 0.7-kp fragment carrying *cpxR* of *E. amylovora* Ea1189	This study
pBBR.egfp.TIR	Cm^r, contains the TIR-*egfp*-T_0 cassette in pBBR1MCS in opposite orientation with respect to *lac* promoter	[2]
pBBR.mdtABC-Pro.egfp	Cm^r, contains a 294-bp fragment carrying the promoter region of *mdtABC*, transcriptional fusion of *mdtABC* with *egfp*	This study
pBBR.mdtUVW-Pro.egfp	Cm^r, contains a 266-bp fragment carrying the promoter region of *mdtUVW*, transcriptional fusion of *mdtUVW* with *egfp*	This study
pET-28a(+)	Km^r, ColE1 origin, expression vector with T7lac promoter, N- and C-terminal His tag	Novagen
pET28a.baeR	Km^r, contains a 0.7-kb fragment carrying *baeR* of *E. amylovora* Ea1189, C-terminal translational fusion with His-tag	This study
pET28a.cpxR	Km^r, contains a 0.7-kb fragment carrying *cpxR* of *E. amylovora* Ea1189, C-terminal translational fusion with His-tag	This study
pBAD24	Ap^r, pBR322 origin, L-arabinose inducible P_{BAD} promoter	[4]
pBAD24.baeR	Ap^r, contains a 0.7-kb fragment carrying *baeR* of *E. amylovora* Ea1189 under control of P_{BAD} promoter	This study
pBAD24.cpxR	Ap^r, contains a 0.7-kb fragment carrying *cpxR* of *E. amylovora* Ea1189 under control of P_{BAD} promoter	This study

[a] Antibiotic resistance: Ap^r, ampicillin; Cm^r, chloramphenicol; Km^r, kanamycin; Sm^r, streptomycin.

References

1. Kovach ME, Phillips RW, Elzer PH, Roop RM, 2nd, Peterson KM: pBBR1MCS: a broad-host-range cloning vector. *BioTechniques* 1994, 16:800-802.
2. Burse A, Weingart H, Ullrich MS: The phytoalexin-inducible multidrug efflux pump AcrAB contributes to virulence in the fire blight pathogen, *Erwinia amylovora*. *Mol Plant-Microbe Interact* 2004, 17:43-54.
3. Hoang TT, Karkhoff-Schweizer RR, Kutchma AJ, Schweizer HP: A broad-host-range Flp-*FRT* recombination system for site-specific excision of chromosomally-located DNA sequences: application for isolation of unmarked *Pseudomonas aeruginosa* mutants. *Gene* 1998, 212:77-86.
4. Guzman LM, Belin D, Carson MJ, Beckwith J: Tight regulation, modulation, and high-level expression by vectors containing the arabinose P_{BAD} promoter. *J Bacteriol* 1995, 177:4121-4130.
