# Supplementary material for: Characterization and regulation of the Resistance-Nodulation-Cell Division-type multidrug efflux pumps MdtABC and MdtUVW from the fire blight pathogen Erwinia amylovora
Source: BMC Microbiol. 2014 Jul 11;14:185. doi: 10.1186/1471-2180-14-185 (PMC4107485; doi:10.1186/1471-2180-14-185)
Supplement: Additional file 7 — Virulence assay on apple rootstock MM106. [file 1471-2180-14-185-S7.pdf]

Additional File 7. Virulence assay on apple rootstock MM 106

Strain	Re-isolated bacterial cells[a]		
	**1 dpi**	**3 dpi**	**7 dpi**
Ea1189	$2.3 \times 10^{7} \pm 1.4 \times 10^{7}$	$2.7 \times 10^{8} \pm 1.3 \times 10^{8}$	$4.7 \times 10^{9} \pm 1.8 \times 10^{9}$
Ea1189.ΔmdtABC	$2.7 \times 10^{7} \pm 1.3 \times 10^{7}$	$4.3 \times 10^{8} \pm 2.1 \times 10^{8}$	$1.5 \times 10^{9} \pm 2.7 \times 10^{8}$
Ea1189.ΔmdtUVW	$2.8 \times 10^{7} \pm 1.6 \times 10^{7}$	$4.3 \times 10^{8} \pm 9.4 \times 10^{7}$	$5.5 \times 10^{8} \pm 2.7 \times 10^{8}$

[a] Bacteria were inoculated by prick technique in the shoot tips with an inoculum of 5×10^{6} CFU/shoot. Establishment of a population (CFU/shoot) of *E. amylovora* Ea1189, *mdtABC*, and *mdtUVW* mutant was determined 1, 3 and 7 days post inoculation (dpi), respectively.
