# Supplementary material for: Characterization and regulation of the Resistance-Nodulation-Cell Division-type multidrug efflux pumps MdtABC and MdtUVW from the fire blight pathogen Erwinia amylovora
Source: BMC Microbiol. 2014 Jul 11;14:185. doi: 10.1186/1471-2180-14-185 (PMC4107485; doi:10.1186/1471-2180-14-185)
Supplement: Additional file 9 — Primers used in this study. [file 1471-2180-14-185-S9.pdf]

Additional File 9. Primers used in this study

Primer	Sequence (5' - 3')	Characteristic(s)
***mdtABC, mdtUVW* knockout**		
mdtABC-A1	AGCAACAGCCAGGGTAGC	used to clone *mdtABC* knockout vector
mdtABC-A2	CCCTATAGTGAGTCGGTACCATACTGAGCAGCGTTCC	used to clone *mdtABC* knockout vector
mdtABC-B1	GGTACCGACTCACTATAGGGCTGGGAAACCAATAGTG	used to clone *mdtABC* knockout vector
mdtABC-B2	ATACCGTGGCAATCGCCG	used to clone *mdtABC* knockout vector
mdtUVW-A1	TCTCTGCGTTATCGTGCTG	used to clone *mdtUVW* knockout vector
mdtUVW-A2	CCCTATAGTGAGTCGGTACCTATTCAGCTTAGCGGCAGC	used to clone *mdtUVW* knockout vector
mdtUVW-B1	GGTACCGACTCACTATAGGGTTCCGATCTCTGCCTTTGC	used to clone *mdtUVW* knockout vector
mdtUVW-B2	AGTGAACAGGGTTAGCAGC	used to clone *mdtUVW* knockout vector
cat_out2	CTTACGTGCCGATCAACG	reverse primer used to confirm insertion of Cm cassette
cat_out3	AGCATTCATCAGGCGGGC	reverse primer used to confirm insertion of Cm cassette
cat_out4	ACAAGGTGCTGATGCCGC	forward primer used to confirm insertion of Cm cassette
cat_out5	GTGATGGCTTCCATGTCG	forward primer used to confirm insertion of Cm cassette
mdtABC_out1	TTCTCACCGTAGCAGCTC	Primer flanking *mdtABC* knockout fragment (used to confirm Cm cassette insertion)
mdtABC_out2	AACGATCAGCCATTGGGC	Primer flanking *mdtABC* knockout fragment (used to confirm Cm cassette insertion)
mdtUVW_out1	TGGCGCTATTTCATGGCG	Primer flanking *mdtUVW* knockout fragment (used to confirm Cm cassette insertion)
mdtUVW_out2	ATTTGCACCTGGCACAGC	Primer flanking *mdtUVW* knockout fragment (used to confirm Cm cassette insertion)
***mdtABC*, *mdtUVW* overexpression**		
mdtABC_SacII	TATCCGCGGATGAAAACACCCCGACG	used to clone *mdtABC* overexpression vectors
mdtABC _ApaI	ATAGGGCCCTCAGCCACAGCGTCC	used to clone *mdtABC* overexpression vectors
mdtABC -P_SacI	CATGAGCTCCTGATTGATAAGTGGATAACGCAG	used to clone upstream region of *mdtABC*
mdtABC -P_SacII	ATACCGCGGTGCTACTCTCCGTGAAATTAAAGCAG	used to clone upstream region of *mdtABC*
mdtUVW_SacI	ATAGAGCTCCGGGGATTATCCTGTCGGGTG	used to clone *mdtUVW* overexpression vectors
mdtUVW_ApaI	TATGGGCCCAGAGCTAAAGGAGTGAGC	used to clone *mdtUVW* overexpression vectors
mdtUVW-P_SacI	CATGAGCTCTTGGCGCTATTTCATGGCG	used to clone the upstream region of *mdtUVW*
mdtUVW-P_SacII	TATCCGCGGAAACCGCGTCGTCTTTTGC	used to clone the upstream region of *mdtUVW*, and used as nested primer for fusion of *mdtUVW* promoter to the *egfp* gene
Transcriptional promoter-egfp fusions		
mdtABC_up	AGGATAATCCGATCGCGCT	used to amplify upstream region of *mdtABC*
mdtABC-P-egfp	CAGCTCCTCGCCCTTGCTCAGCATCCTGGGTGCTACTCTCCG	used to amplify upstream region of *mdtABC* (contains a 24-nt extension that is homologous to the start of the *egfp* gene)
mdtUVW_up	GCGATTAAAACCGGTACTGC	used to amplify upstream region of *mdtUVW*
mdtUVW-P-egfp	CAGCTCCTCGCCCTTGCTCAGCATCCGACAGGATAATCCCCG	used to amplify upstream region of *mdtUVW* (contains a 24-nt extension that is homologous to the start of the *egfp* gene)

egfp-ATG	ATGCTGAGCAAGGGCGAG	used to amplify the *egfp* gene flanked downstream by translational stop codons in all three reading frames and the transcriptional terminator t0 from phage λ
egfp-Cm	TACGCAAACCGCCTCTCC	used to amplify the *egfp* gene flanked downstream by translational stop codons in all three reading frames and the transcriptional terminator t0 from phage λ
mdtABC-P_SacII	TATCCGCGGTATGATTCTCCTGATTGATAAGTGG	nested primer used for fusion of the *mdtABC* promoter to the *egfp* gene
uidA-t0_KpnI	TATGGTACCAACGGTGGTATATCC	nested primer used for fusion of a promoter region to the *egfp* gene
baeR, cpxR overexpression		
baeR_SacII	ATACCGCGGATGAACCAGATCCCCGCCAC	used to clone *baeR* overexpression vector
baeR_ApaI	GTAAAGCGGGGGCCCGGC	used to clone *baeR* overexpression vector
cpxR(HindIII)_fwd	AAGCTTCATGTTGCCGATACCATCA	used to clone *cpxR* overexpression vector, and used to clone *cpxR* into C-terminal His-tag protein expression vector
cpxR(EcoRI)_rev	GAATTCATGAATAAGATCTTATTGGTTGACGAC	used to clone *cpxR* overexpression vector, and used to clone *cpxR* into C-terminal His-tag protein expression vector
Electrophoretic mobility shift assay		
mdtABC-P-fwd	TGATTCTCCTGATTGATAAGTGG	used to amplify the *mdtABC* promoter region
mdtABC -P-rev-Cy5	ACCGTCAAGGCGATAAGG	used to amplify the *mdtABC* promoter region, Cy5 labeled
mdtUVW-P-fwd	TCTACGGTCACGGTTAAG	used to amplify the *mdtUVW* promoter region
mdtUVW-P-rev-Cy5	TAAACAACACCCGACAGG	used to amplify the *mdtUVW* promoter region, Cy5 labeled
tolC-in-fwd	TCGAGCCTGGCTTCACTC	used to amplify a fragment from the *tolC* gene
tolC-in-rev-Cy5	TGGTTGCTGGCTGACTGG	used to amplify a fragment from the *tolC* gene, Cy5 labeled
baeR_NcoI	TATCCATGGACCAGATCCCCGCCACTC	used to clone *baeR* into C-terminal His-tag protein expression vector
baeR_EcoRI	ATTGAATTCGGGATCAGACGACAGCCATC	used to clone *baeR* into C-terminal His-tag protein expression vector
Quantitative RT-PCR		
mdtA_RT_fwd	AGTATTTGTCCGGGCTGGG	
mdtA_RT_rev	TAAAGCGACCTCGTACGGG	
mdtU_RT_fwd	TGTTCTGGCGCTTATGGCC	
mdtU_RT_rev	AATAACCGTTCCCAGCGCG	
baeR_RT_fwd	ATGAGTACCAGCAGCACGG	
baeR_RT_rev	TGACGTGCGATTGCTGGTC	
cpxR_RT_fwd	TTCTGGTTGCTGGTGATGG	
cpxR_RT_rev	CGTCTGATGTTGTTGACGC	
recA_RT_fwd	TAAGGGCTCCATCATGCGC	
recA_RT_rev	ACCTGCAAAGTCAGGGTGG	
